# Genomic Epidemiology and Evolution of Scallion Mosaic Potyvirus From Asymptomatic Wild Japanese Garlic

**DOI:** 10.3389/fmicb.2021.789596

**Published:** 2021-12-08

**Authors:** Kazusato Ohshima, Shusuke Kawakubo, Satoshi Muraoka, Fangluan Gao, Kanji Ishimaru, Tomoko Kayashima, Shinji Fukuda

**Affiliations:** ^1^Department of Biological Resource Science, Faculty of Agriculture, Saga University, Saga, Japan; ^2^Institute of Wild Onion Science, Saga University, Saga, Japan; ^3^The United Graduate School of Agricultural Sciences, Kagoshima University, Kagoshima, Japan; ^4^Institute of Plant Virology, Fujian Agriculture and Forestry University, Fuzhou, China; ^5^Department of School Education Course, Faculty of Education, Saga University, Saga, Japan; ^6^Saga University Center for Education and Research in Agricultural Innovation, Faculty of Agriculture, Saga University, Saga, Japan

**Keywords:** scallion mosaic virus, potyvirus, turnip mosaic virus phylogenetic group, evolution, epidemiology, recombination, asymptomatic, wild garlic plants

## Abstract

Scallion mosaic virus (ScaMV) belongs to the turnip mosaic virus phylogenetic group of potyvirus and is known to infect domestic scallion plants (*Allium chinense*) in China and wild Japanese garlic (*Allium macrostemon* Bunge) in Japan. Wild Japanese garlic plants showing asymptomatic leaves were collected from different sites in Japan during 2012–2015. We found that 73 wild Japanese garlic plants out of 277 collected plants were infected with ScaMV, identified by partial genomic nucleotide sequences of the amplified RT-PCR products using potyvirus-specific primer pairs. Sixty-three ScaMV isolates were then chosen, and those full genomic sequences were determined. We carried out evolutionary analyses of the complete polyprotein-coding sequences and four non-recombinogenic regions of partial genomic sequences. We found that 80% of ScaMV samples have recombination-like genome structure and identified 12 recombination-type patterns in the genomes of the Japanese ScaMV isolates. Furthermore, we found two non-recombinant-type patterns in the Japanese population. Because the wild plants and weeds may often serve as reservoirs of viruses, it is important to study providing the exploratory investigation before emergence in the domestic plants. This is possibly the first epidemiological and evolutionary study of a virus from asymptomatic wild plants.

## Introduction

It is important to investigate the virus populations in wild host plants before they spill over and start infecting domestic plants (Ohshima et al., 2010; Nguyen et al., 2013; Gibbs et al., 2015) because the wild plants and weeds may often serve as reservoirs of viruses. For viruses to infect new hosts, their genomes may need to be modified through mutation and recombination, which can occur within either the old or new hosts (Ohshima et al., 2007; Gibbs et al., 2015).

There have been many studies of the molecular epidemiology and evolution of viruses infecting domestic plants (Ohshima et al., 2002; Seo et al., 2009; Lefeuvre et al., 2010; Ohshima et al., 2016b; Fuentes et al., 2019; Gao et al., 2020), but fewer for those infecting wild plants (Prendeville et al., 2012; Malmstrom and Alexander, 2016; García-Arenal and Zerbini, 2019). Moreover, there have been few studies of viruses from asymptomatic wild plants, because these pathogens do not have immediate impacts on agriculture (Roossinck, 2015; Hillman et al., 2018; Martínez-Marrero et al., 2020). Most of the viruses that infect domestic plants in modern agriculture possibly originated and emerged from wild plants before or during the development of agriculture (Nguyen et al., 2013; Kawakubo et al., 2021). Therefore, although virus spillover is a complex and poorly understood process, characterizing the virus populations in wild host plants is very important.

Potyviruses are plant viruses that cause significant damage to a wide range of monocotyledonous and dicotyledonous plant species across the globe (Gibbs and Ohshima, 2010; Wylie et al., 2017; Gibbs et al., 2020). These viruses are transmitted by aphids in a non-persistent, non-circulative manner. Potyviruses have flexuous filamentous particles 700–750nm long, and each contains a single copy of the genome. The genome is a single-stranded, positive-sense RNA molecule of approximately 10,000 nucleotides with one major open reading frame (ORF) that is translated into one large polyprotein and with a small overlapping ORF (Chung et al., 2008).

Potyviruses have been divided into several phylogenetic groups (Gibbs and Ohshima, 2010; Wylie et al., 2017). The turnip mosaic virus (TuMV) group is one of the best studied in terms of molecular evolution (Ohshima et al., 2002; 2016a,c,d, 2018; Yasaka et al., 2017; Kawakubo et al., 2021). This group includes TuMV, Japanese yam mosaic virus (JYMV; Fuji and Nakamae, 1999; 2000), narcissus late season yellows virus (NLSYV), narcissus yellow stripe virus (NYSV; Wylie et al., 2014; Ohshima et al., 2018), wild onion symptomless virus (WoSV; Ohshima et al., 2016a), and scallion mosaic virus (ScaMV; Chen et al., 2002). Among these viruses, JYMV, narcissus viruses, and ScaMV infect both monocotyledonous wild and domestic plants, whereas WoSV is known to infect only monocotyledonous wild *Allium* sp. TuMV mostly infects dicotyledonous domestic Brassicaceae plants but possibly originated from monocotyledonous wild orchids 700years ago (Gera et al., 1997; Nguyen et al., 2013; Kawakubo et al., 2021).

Wild Japanese garlic (Chinese garlic or no-biru, a species of wild onion) is known to be edible but is not yet established as a crop and is the most widely distributed wild *Allium* plant in Japan. It is widespread in East Asian countries such as China or Korea (Wu et al., 1994; Schoch et al., 2020). Onions and their relatives (*Allium* sp.) are monocotyledons and are susceptible to potyviruses including onion yellow dwarf virus (OYDV), leek yellow stripe virus (LYSV), shallot yellow stripe virus (SYSV), ScaMV, and some isolates of TuMV (Brunt et al., 1996). ScaMV was first reported from domestic scallion plants (*Allium chinense*) in China (Chen et al., 2002), and then from wild Japanese garlic (*A. macrostemon* Bunge) in Japan (Ohshima et al., 2016c).

In this study, wild Japanese garlic plants with asymptomatic leaves were collected from different sites on riverbanks and fields around Japan because the wild plants may often serve as reservoirs of viruses. We inferred its phylodynamics by analyzing the full genomic sequences of a representative population of ScaMV. Our results reveal the genomic epidemiology and evolution of ScaMV.

## Materials and Methods

### Plant Specimens

**Figure 1:**
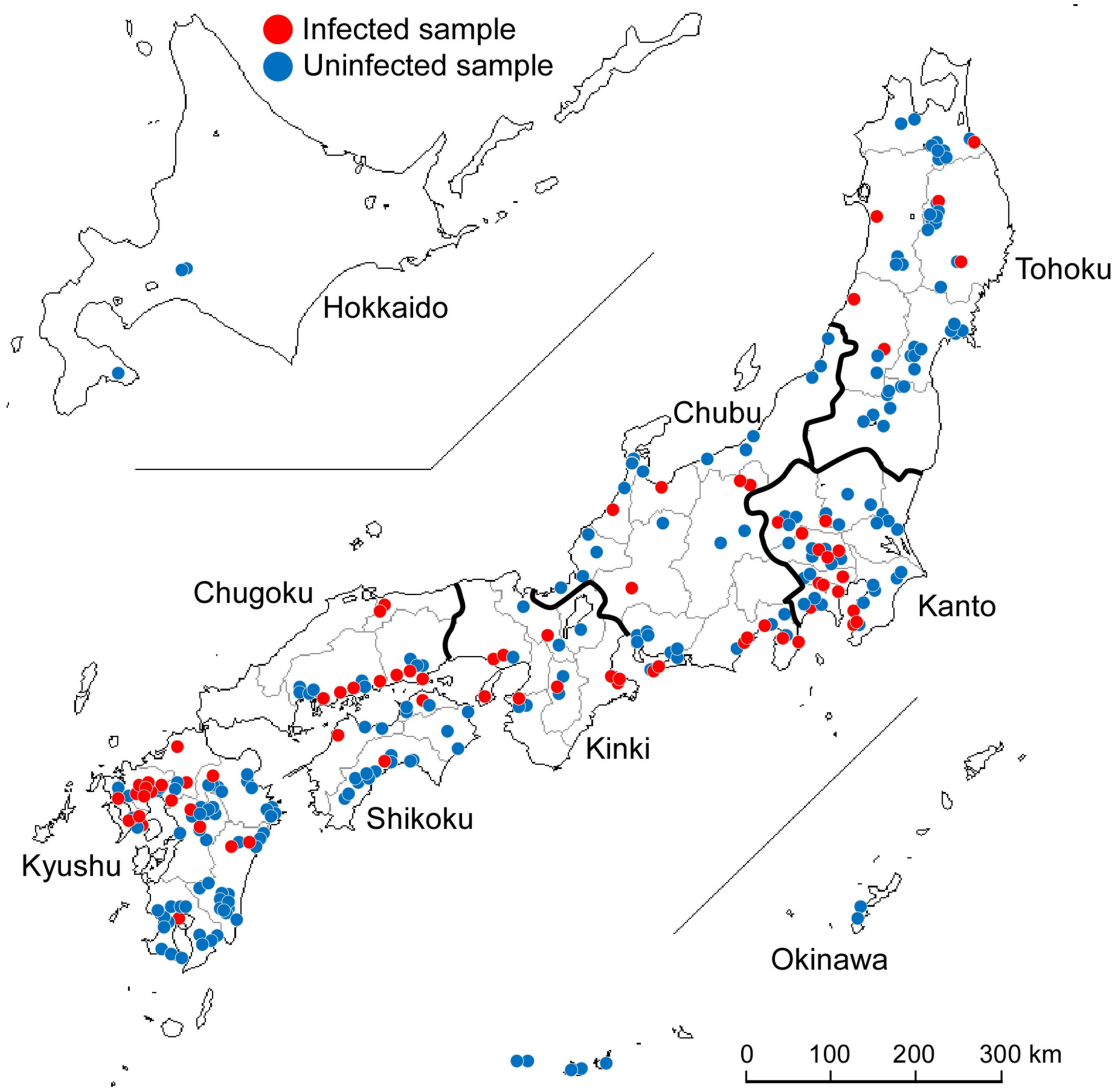
Collection of wild Japanese garlic (*Allium macrostemon* Bunge) plants showing striped and asymptomatic leaves from different sites in Japan during 2012–2015. The map was obtained from http://www.craftmap.box-i.net/

A total of 277 wild Japanese garlic plants (*A. macrostemon* Bunge) were collected from different sites on the banks of rivers and fields around Japan during the winter and spring seasons of 2012–2015 (Figure 1). We searched for wild Japanese garlic plants on foot and by car. Those plant leaves were mostly asymptomatic and rarely showed mild yellowing and striping. Details of the plants, their place of origin, site types, and years of isolation are shown in Supplementary Tables S1,S2.

### Detection, Cloning, and Sequencing of Virus Genomes

Because the sap from the collected wild Japanese garlic plants did not induce local lesions on *Chenopodium amaranticolor*, *C. quinoa*, *Nicotiana benthamiana*, *N. tabacum*, or some *Allium* plants, we were unable to clone the Japanese garlic plant-infected viruses biologically. Therefore, the viruses were directly identified from the collected asymptomatic Japanese garlic leaves by reverse transcription and polymerase chain reaction (RT-PCR) using potyvirus-specific primer pairs which were expected to amplify all potyviruses (Supplementary Table S3), and by the partial sequencing of the cloned RT-PCR products as described below. The total RNAs were extracted using Isogen RNA extraction reagent (Nippon Gene, Tokyo, Japan) from the leaves of wild Japanese garlic (Supplementary Table S2). The RNAs were reverse-transcribed by PrimeScript II Moloney murine leukemia virus reverse transcriptase (Takara Bio, Shiga, Japan), and the potyvirus cDNAs were amplified using high-fidelity Prime STAR GXL DNA Polymerase in PrimeScript II High Fidelity One-Step RT-PCR Kit (TaKaRa Bio, Shiga, Japan). The RT-PCR conditions were: 45°C for 10min for RT, one cycle of 94°C for 2min, and 40cycles of 98°C for 10s, 45°C for 15s, and 68°C for 35s. RT-PCR products of approximately 2,100bp were amplified from Japanese garlic leaves using potyvirus-specific primer pairs (Ohshima et al., 2016c,d, Supplementary Table S3), POTYNIbNOT4P (5′- GGGGCGGCCGCATATGGGGTGAGAGAGG TNTGYG TNGAYGAYTTYAAYAA −3′) and Tu3T9M (5′- GGGGCG GCCGCT_15_-3′) for virus genome amplification. This primer pair amplifies the most of potyvirus genomes. The amplified cDNAs were separated by electrophoresis in agarose gels and purified using a QIAquick Gel Extraction kit (Qiagen K.K., Tokyo, Japan). The RT-PCR products were cloned into NotI site of plasmid pZErO-2 (Invitrogen-ThermoFisher com, Tokyo, Japan). At least 10 independent clones from each virus-infected Japanese garlic plant were obtained.

The nucleotide sequences (approximately 600–700bp) of parts of the amplified fragments from all clones from 22 isolates were first determined using POTYNIB5P primer (5′- CGCATATGGGGTGAGAGAGG- 3′), a part of POTYNIbNOT4P (underlined), using a BigDye Terminator v3.1 Cycle Sequencing Ready Reaction kit (Applied Biosystems, Foster City, CA, United States) and an Applied Biosystems Genetic Analyzer DNA model 3130. Because large numbers of clones were obtained, we first aimed to identify the viruses infecting each Japanese garlic plant and select clones of 600–700bp sequences to obtain the sequence of the complete coat protein (CP) coding region and the full genomic sequence of some virus isolates. As a result, BLAST searches showed that all of the cloned sequences were closely related to the sequences of ScaMV. The sequences of clones from the middle of the nuclear inclusion b (NIb) coding region to the 3′ end region (NIb/3′ end region) of the viral genomes were also determined in both directions using ScaMV-specific primers (Supplementary Table S3) and primer walking, and then, we confirmed that all of the clones were derived from ScaMV.

Several fragments covering the full genomic regions of ScaMV were amplified by RT-PCR (Supplementary Figure S1). For cloning the partial genomic regions, the three fragments, from the 5′ end to the nuclear inclusion a proteinase protein (NIa-Pro) coding region (5′ end/NIa-Pro region), from cylindrical inclusion protein (CI) coding region to 3′ non-coding region (NCR; CI/3′ NCR region), and the NIb/3′ end region, were amplified by RT-PCR using appropriate primers designed from sequences obtained in the present study and from the TuMV phylogenetic group viruses obtained in the public databases. For direct sequencing of RT-PCR products, the five or more fragments, from the 5′ end to third protein (P3) or the first 6kDa protein (6K1) coding region (5′ end/P3 or 5′ end/6K1 region), P3 to NIa-Pro coding region (P3/NIa-Pro region), from CI coding region to 3’ NCR region (CI/3′ NCR region), and the NIb/3′ end region, were amplified. Some genomic regions were also amplified to confirm the sequences obtained by cloning or direct sequencing methods.

The RT-PCR products were cloned into NotI site of plasmid pZErO-2 or used for direct sequencing. At least three independent clones for each fragment were obtained. The overlapping regions between RT-PCR products were at least 500 nucleotides (Supplementary Figure S1), and clones and fragments that had no mismatch in the overlapping regions were assembled to obtain full genomic sequences. These overlappings ensure no artificial recombination events in the ScaMV genomes during the sequencing and recombination analyses. The nucleotide sequences of clones were determined in both directions by primer walking using more than 40 primers (Supplementary Table S3) because of the variation between the sequences of each isolate. After we confirmed almost no mismatches between the clones and between the overlapping fragments using 16 isolates (Supplementary Table S2), we then directly sequenced the RT-PCR products of the partial or full genomic sequences of ScaMV isolates. Most of the primers used in this study were synthesized with reference to the full genomic sequences of six TuMV phylogenetic group viruses and potyviruses. Sequence data were assembled using BioEdit version 5.0.9 (Hall, 1999).

### Network Communities and Recombination Analysis

Standard phylogenetic approaches were not feasible for our full-length dataset because ignoring non-vertically inherited regions can lead biased inference of topology (Schierup and Hein, 2000) and recombination occurs frequently in potyviruses (Ohshima et al., 2007). To understand the complexity of our entire set of full-genomic sequences, we used *k*-mer distances (Vinga and Almeida, 2003) to represent the network communities of ScaMV. The frequencies of all possible hexamers in each sequence were counted to obtain a pairwise distance matrix using a Python script (kindly supplied by Dr. Art F. Y. Poon) as described by Olabode et al. (2019). The resulting matrix was converted into an undirected adjacency graph with a threshold 0.91 using the *igraph* package (Csardi and Nepusz, 2006) and then visualized with Graphviz (Gansner and North, 2000).

We aligned all 64 ScaMV amino acid [polyprotein; major ORF] sequences with those of the TuMV phylogenetic group viruses as outgroup taxa using CLUSTAL X version 2 (Larkin et al., 2007) with TRANSALIGN (Weiller, 1999) to maintain the degapped alignment of the encoded amino acids, and then reverse-translated to nucleotides to form complete polyprotein-coding sequences. The outgroup taxa to align ScaMV genomic sequences were three JYMV (Fuji and Nakamae, 1999; 2000; Lan et al., 2015), two TuMV (Nguyen et al., 2013), three NLSYV, three NYSV (Chen et al., 2006; Lin et al., 2012; Wylie et al., 2014; Ohshima et al., 2018), and one WoSV (Ohshima et al., 2016a).

The aligned 5′ and 3′ NCR sequences were then reassembled with both ends of the polyprotein-coding sequences to form nearly complete genomic sequences of 9,294 nucleotides, excluding the 26 nucleotides that were used to design the primer for RT-PCR amplification. Those were assessed for evidence of recombination, especially for recombination sites both in the polyprotein and in the NCRs. Firstly, putative recombination sites in all sequences were identified using RDP (Martin and Rybicki, 2000), GENECONV (Sawyer, 1999), BOOTSCAN (Salminen et al., 1995), MAXCHI (Smith, 1992), CHIMAERA (Posada and Crandall, 2001), and SISCAN (Gibbs et al., 2000) programs implemented in the RDP4 version 100 software package (Martin et al., 2015) and also the original SISCAN version 2 program (Gibbs et al., 2000). First, we checked for incongruent relationships using the programs implemented in RDP4. These analyses were done using default settings for the different detection programs and a Bonferroni corrected *p*-value cutoff of 0.05 or 0.01. All isolates that had been identified as likely recombinants by the programs in RDP4, supported by three different methods with an associated value of *p*<10^4^ (i.e., the most likely recombination sites), were re-checked using the original SISCAN version 2 with all nucleotide sites. We checked 100- and 50-nucleotide sliding window of all sequences for evidence of recombination using these programs. For convenience, we refer to them as the “parental isolates” of recombinants. Second, ScaMV sequences were also aligned without outgroup sequences and directly checked for evidence of recombination using the programs. Additionally, we reconstructed phylogenetic trees using the successive partial overlapping sequences in the genomes and evaluated the topologies of the clustering of each group between the recombination sites. This provides a means of clarifying the recombination sites of unknown parental sequences. Finally, GARD (Kosakovsky Pond et al., 2006) and SIMPLOT version 3.5.1 (Lole et al., 1999) with a window length of 200 and step size of 20 were used to assess evidence of the recombination sites.

### Estimating the Evolutionary Timescale

We estimated the evolutionary timescale for ScaMV. The dataset was analyzed using the Bayesian method implemented in BEAST v1.10.4 (Suchard et al., 2018) under a lognormal relaxed clock model and a Bayesian skyline coalescent tree prior (marginal likelihoods estimated by the path sampling), with 10 replicates in which the sampling dates were randomized among the sequences.

### Spatial Diffusion of Each Recombination-Type Pattern

We applied a continuous phylogeographic model (Lemey et al., 2010) to infer the spatial diffusion of each non-recombinant- and recombination-type pattern of ScaMV population in Japan. The recombination-type patterns that comprised fewer than three isolates were not included, because they were under the minimum number of taxa for phylogenetic analysis. The polyprotein-coding sequences of each isolate were assigned with two-dimensional geographic coordinate (i.e., latitude and longitude) and analyzed using BEAST v1.10.4 (Suchard et al., 2018). Markov chain Monte Carlo analyses were run for 100 million steps each, sampled at every 10,000 steps across three independent Markov chains. We used Tracer v1.7.1 (Rambaut et al., 2018) to check for convergence and satisfactory mixing, based on the effective sample size exceeding 200 for each parameter. In this analysis, the phylogeographic reconstruction was carried out using a relaxed random walk diffusion model that draws branch-specific rate scalers from a gamma distribution. For visualizing the inferred spatial distribution of each lineage, we processed 1,000 post-burn-in sampled trees in SpreaD3 version 0.9.7 (Bielejec et al., 2016). The estimated locations of each lineage were plotted with statistical uncertainty (95% credible intervals).

### Phylogenetic Analysis and Diversity

We aligned the polyprotein-coding sequences of the 64 ScaMV isolates, together with those of three NYSV, three NLSYV, three JYMV, one WoSV, and two TuMV isolates, as outgroup taxa, as described above using CLUSTAL X version 2 with TRANSALIGN. Phylogenetic relationships were inferred using the Neighbor-Net method in SPLITSTREE v4.11.3 (Huson and Bryant, 2006) and maximum likelihood implemented in PhyML version 3 (Guindon and Gascuel, 2003) using the general time-reversible substitution model with gamma-distributed site rates (GTR+I+Γ4). The best-fit model of nucleotide substitution for each dataset was determined using jModeltest version 2.1.2 (Darriba et al., 2012). For maximum-likelihood analyses, branch support was evaluated by the bootstrap method based on 1,000 pseudoreplicates. The inferred trees were displayed by TREEVIEW (Page, 1996). The nucleotide and amino acid diversities of ScaMV were estimated using MEGA version 7 (Kumar et al., 2016). We used EMBOSS Needle (Rice et al., 2000 ^1^) and Sequence Demarcation Tool (SDT) v1.2 (Muhire et al., 2014) to estimate nucleotide identity between 64 isolates. The degree of mutational saturation in the ORF sequences was evaluated using the Iss statistic in DAMBE version 6.4.81 (Xia, 2017).

## Results and Discussion

### Molecular Characteristics

A total of 277 wild Japanese garlic plants were collected in Japan during 2012–2015 (Supplementary Tables S1,S2). We found that the garlic plants were easier to collect in southern area of Japan but harder in Hokkaido Island, probably because the southern area might be suitable for the conditions of growing wild Japanese garlic plants. We collected mostly asymptomatic plants in the present study, because in our preliminary investigations we noticed that some asymptomatic plants were also infected with ScaMV.

The wild Japanese garlic samples collected were firstly checked for infection by potyviruses and ScaMV by RT-PCR, using the potyvirus-specific primer pair of POTYNIbNOT4P and Tu3T9M, and sequencing of the RT-PCR products directly or after cloned into NotI site of plasmid pZErO-2 (Ohshima et al., 2016c,d). Of those checked, 73 plants (26%) were found to be infected with ScaMV: none from Hokkaido, six from Tohoku, 14 from Kanto, 12 from Chubu, nine from Kinki, nine from Chugoku, three from Shikoku, 20 from Kyushu, and none from Okinawa (Figure 1; Supplementary Tables S1,S2). Sequences of other potyviruses, including OYDV, SYSV, and LYSV, were not found in the wild Japanese garlic plants (data not shown).

We attempted to inoculate the sap of some of ScaMV-infected wild Japanese garlic plants to *C. amaranticolor*, *C. quinoa*, or other test plants that might show local lesions to investigate the plants suitable for biological cloning of ScaMV. Unfortunately, none of these plants induced any local lesions. Therefore, we cloned and sequenced the plasmid cloned sequences from RT-PCR products amplified using several potyvirus- or ScaMV-specific primer pairs (Supplementary Table S3) to find mixed infections of different isolates of ScaMV. More than 48 clones covering the full genomic sequences of 16 ScaMV isolates were determined (approximately 400,000 nucleotides in total). We did not find any mismatches in the genomic sequences of the cloned plasmid sequences of the identical genomic regions in the ScaMV full genomes of 16 isolates. This shows that most of the wild Japanese garlic plants in Japan were each infected with single ScaMV isolate. Therefore, we decided to sequence directly the genomes of the remaining 47 isolates using the RT-PCR products.

We determined the full genomic sequences of 63 ScaMV isolates. All of the genomes of Japanese isolates of ScaMV were 9,296–9,300 nucleotides in length, excluding 5′-end 26nt primer sequence. The polyproteins of most isolates are 9,003 nucleotides in length except one isolate WOTC465 with 9,006 nucleotides. All the regions encoding HC-Pro, P3, PIPO, the second 6kDa protein (6K2), VPg, NIa-Pro, NIb, and CP proteins were 1,371, 1,062, 198, 156, 1932, 159, 576, 729, 1,551, and 834 nucleotides, respectively, and the P1 coding region was 633 nucleotides for most isolates except the WOTC465 isolate with 636 nucleotides. All of the motifs reported for different potyvirus encoded proteins were found. The new genomic sequences determined in this study are available in DDBJ/EMBL/GenBank databases with Accession numbers LC651456–LC651518. These 63 genomic sequences, along with one sequence from a Chinese isolate from GenBank (Chen et al., 2002, accession no NC_003399), were included in our analyses.

### Distribution of Non-recombinants and Recombinants

**Figure 2:**
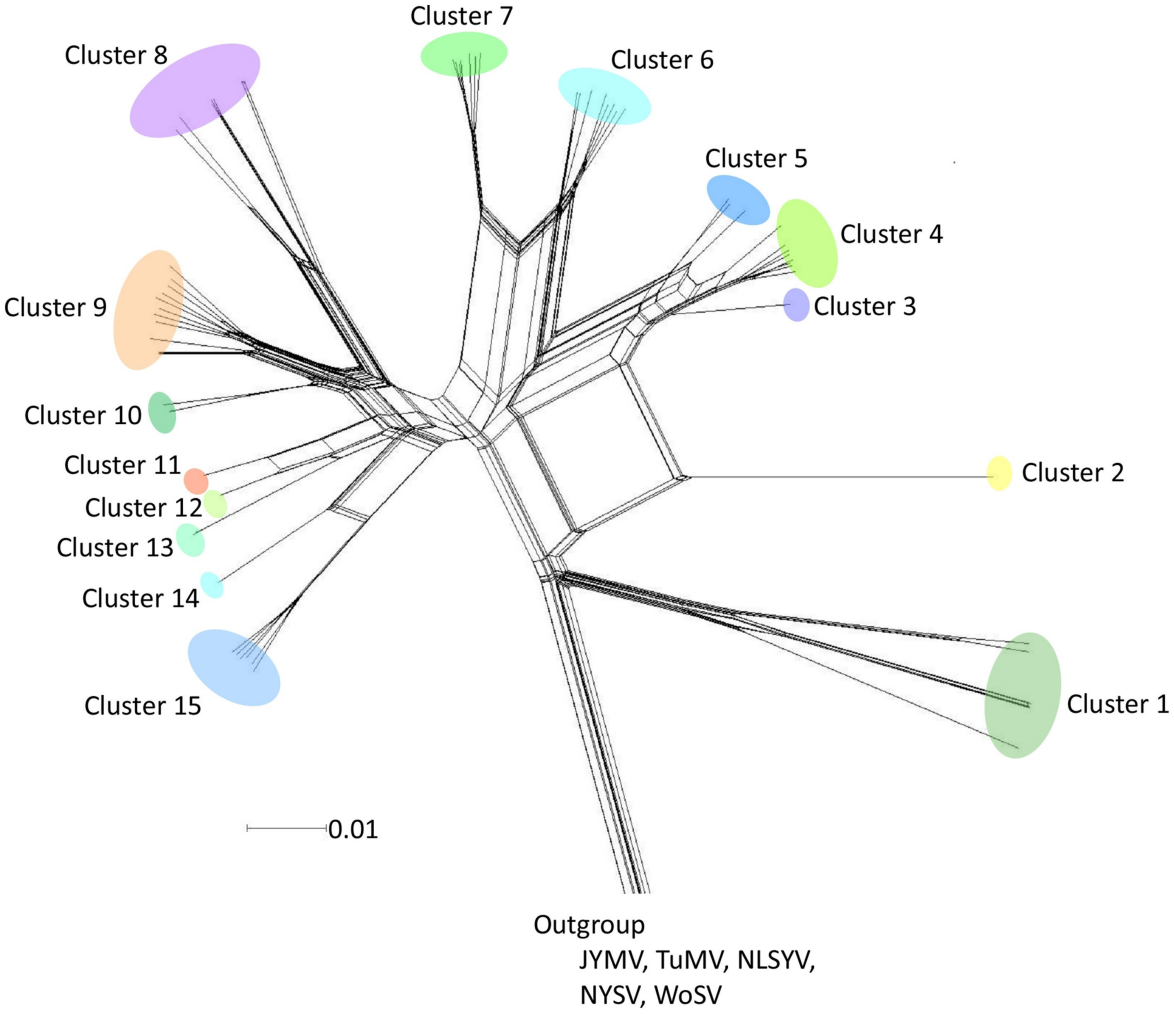
Split-decomposition phylogenetic networks. Neighbor-Net tree inferred from polyprotein-coding sequences of 64 scallion mosaic virus (ScaMV) isolates. Three sequences of Japanese yam mosaic virus (JYMV; Fuji and Nakamae, 1999, 2000; Lan et al., 2015), two turnip mosaic virus (TuMV; Nguyen et al., 2013), three narcissus late season yellows virus (NLSYV), three narcissus yellow stripe virus (NYSV;Chen et al., 2006; Lin et al., 2012; Wylie et al., 2014; Ohshima et al., 2018), and one wild onion symptomless virus (WoSV; Ohshima et al., 2016a) were used as outgroup taxa. The scale bar indicates genetic distance.

A phylogenetic network was inferred using Neighbor-Net (NN) from the polyprotein-coding sequences of 8,949 nucleotides (Figure 2). The analysis showed reticulated phylogenetic networks with at least 15 clusters, reflecting conflicts in the phylogenetic signal that were presumably due to the presence of recombinant sequences. Although there were small-scale geographical groupings of isolates in the networks, there was no clear congruence between the relationships among the isolates and their provenance.

**Figure 3:**
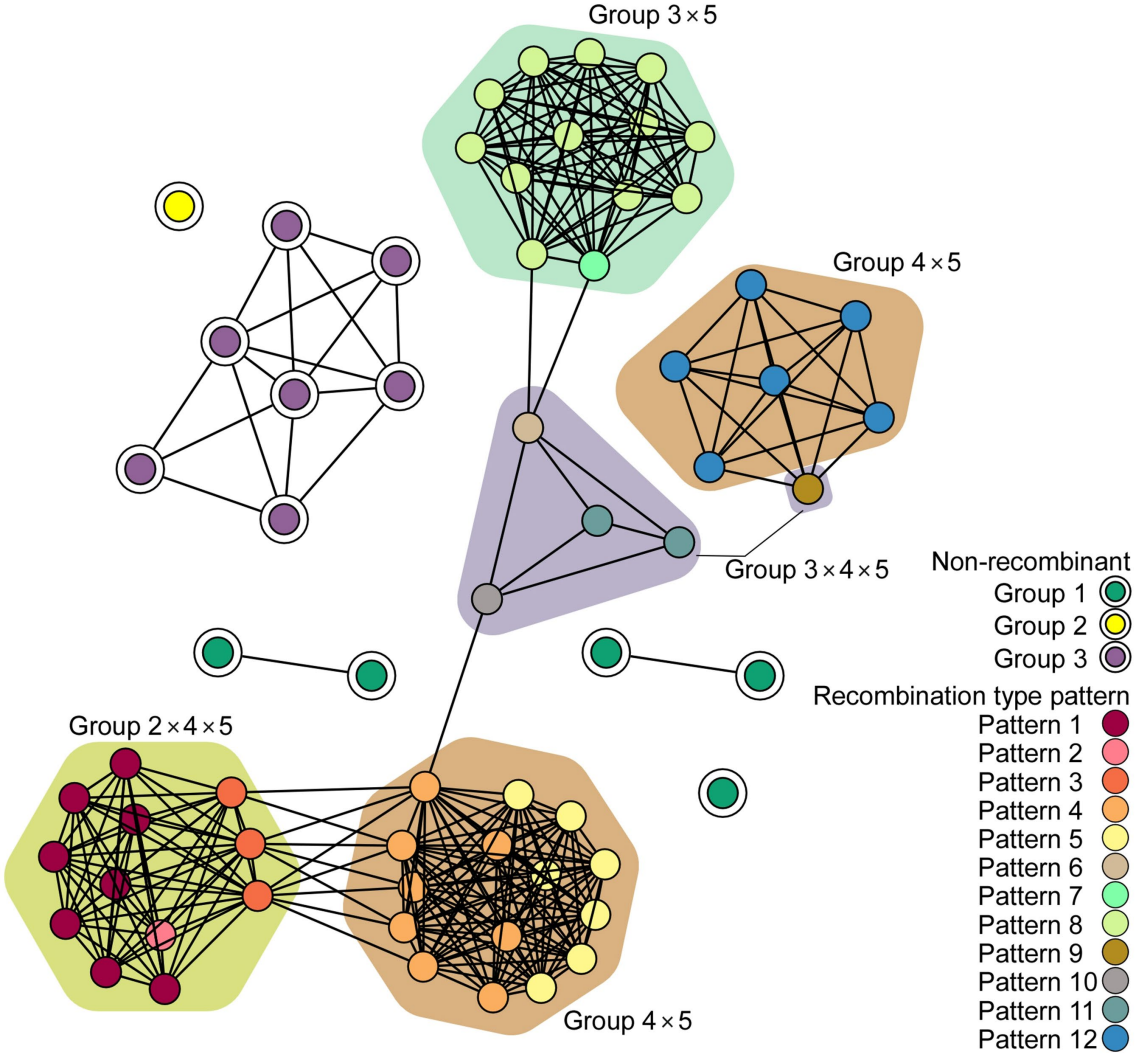
Network diagram of scallion mosaic virus (ScaMV) genomic sequences. Each node represents a ScaMV genome, and each edge indicates that respective node has a pairwise *k*-mer distance under the threshold (0.91). Double circled nodes represent non-recombinant ScaMV genomes and colored by their phylogenetic group, whereas non-circled nodes represent recombinant ScaMV genomes. The polygon color represents the combination of phylogenetic group in their parental sequences in each recombinant.

The network communities of ScaMV were reconstructed using 64 genome sequences, using *k*-mer distances (Figure 3). The network reveals the existence of several major recombinant subpopulations, appearing as closely related clusters. However, none of the recombinant genomes shared communities with non-recombinant genomes, suggesting the importance of undiscovered phylogenetic groups. Further sampling of ScaMV isolates will help to fill these gaps and to clarify the evolutionary characteristics of the virus.

**Figure 4:**
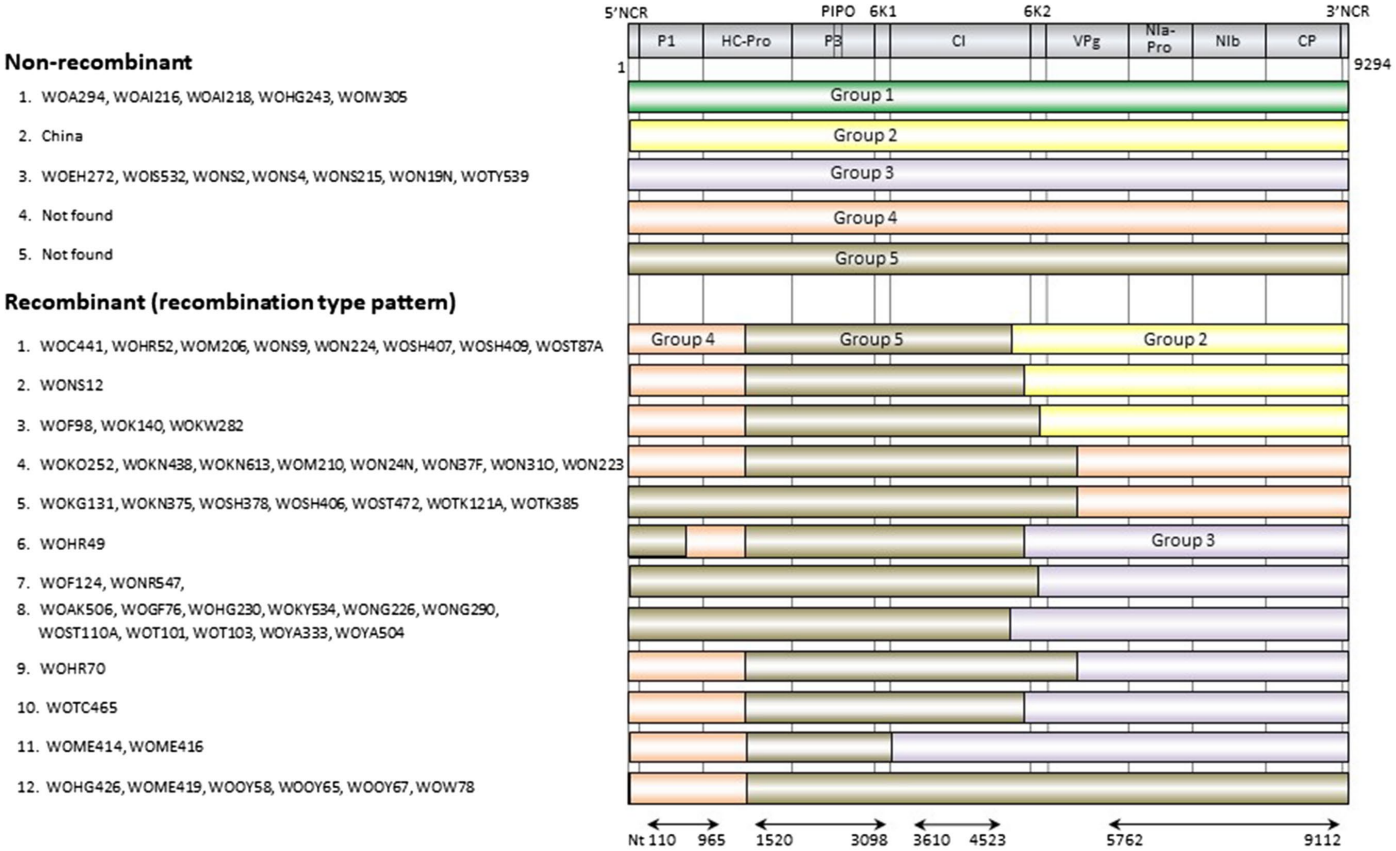
Recombination genome map of scallion mosaic virus genomes of the isolates. Vertical solid lines show estimated approximate recombination sites. The nucleotide positions are shown relative to the 5' end of the genome using the numbering of the aligned and degapped sequences used in the present study, nearly complete genomic sequences of 9,294nt long excluding the 5' end of 26 nucleotides that were used to design the primer for RT-PCR amplification (see Materials and Methods). The horizontal arrows show the regions used to infer phylogenetic trees from non-recombinant sequences (Figure 5).

The genomic sequences of 63 Japanese and one Chinese isolate were assessed for evidence of recombination. We found 11 unequivocal recombination sites in the genomes of 51 Japanese isolates (Figure 4; Supplementary Table S4) with these sites were located in the P1, 6K1, CI, 6K2, and VPg coding regions. We further assessed evidence of the recombination sites using SIMPLOT and GARD softwares, and several phylogenetic trees of the successive partial overlapping sequences in the genomes. The four phylogenetic trees using the partial genomic regions between the major recombination sites were inferred using nt 110–965, nt 1,520–3,098, nt 3,610–4,523, and nt 5,672–9,112 (Figure 4). Considering the topologies of the clustering in each group, we found one tentative recombination site in HC-Pro coding region. For instance, 51 isolates fell into Group 5 in nt 1,520–3,098 tree, whereas the isolates split into Groups 4 and 5 in the 110–965 tree. This indicates that there is a recombination site between nt 965 and 1,520 of HC-Pro coding region. The recombination site was not clearly identified by RDP4 in the present study, but the position of the site will become clearer when the parental sequences of Groups 4 and 5 are identified through further studies. We also used GARD and SIMPLOT softwares to assess evidence of the recombination sites (Supplementary Figure S2). The results also showed the evidence of recombination at identical sites to those of the RDP4 and phylogenetical analyses. Although the SIMPLOT and GARD softwares identified one tentative recombination site in the middle of CP coding region, this site was unclear from the topologies of the 5′ and 3′ side phylogenetic trees (data not shown).

**Figure 5:**
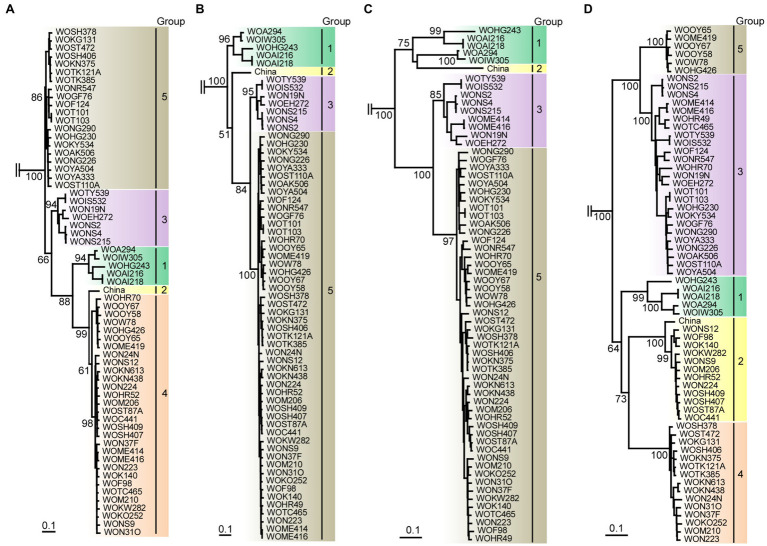
Maximum-likelihood tree showing the relationships of the four partial genomic sequences of scallion mosaic virus (ScaMV) isolates and the viruses in TuMV phylogenetic group. For partial genomic regions, **(A)** nt 110–965, **(B)** nt 1,520–3,098, **(C)** nt 3,610–4,523, and **(D)** nt 5,762–9,112 were used to construct trees after removing gaps from aligned sequences. Three homologous sequences of JYMV (Fuji and Nakamae, 1999, 2000; Lan et al., 2015), two TuMV (Nguyen et al., 2013), three NLSYV and three NYSV (Chen et al., 2006; Lin et al., 2012; Wylie et al., 2014; Ohshima et al., 2018), and one WoSV (Ohshima et al., 2016a) were used as outgroup taxa; however, those are not shown. Numbers at each node indicate bootstrap percentages based on 1,000 pseudoreplicates. (Only percentages greater than and equal to 50 are shown.) Horizontal branch lengths are drawn to scale with the bar indicating 0.1nt substitution per site. The inferred trees were displayed by TreeView (Page, 1996).

There are at least five non-recombinants (parental sequences of Groups 1–5) in the ScaMV population, but only two and one parental non-recombinants were found in the Japanese and Chinese population, respectively (Figure 4). Since we collected ScaMV throughout Japan, the remaining parental non-recombinants that were not found in Japan might be distributed in East Asian countries other than Japan. In total, we identified 12 recombination-type patterns in genomes from the Japanese population, with 51 out of 64 (80%) ScaMV isolates showing evidence of recombination.

The recombination sites in the genomes of potyviruses are well studied. For instance, inter- and intralineage recombinants are common in natural populations of TuMV (Tan et al., 2004). Furthermore, the P1, NIa-Pro, and VPg coding regions in the TuMV genomes are known as recombination hotspots (Ohshima et al., 2007). In other potyviruses, the junction of HC-Pro and P3 coding regions (Green et al., 2017) and from the CI to NIa coding regions is also known as recombination hotspots (Seo et al., 2009; Tugume et al., 2010). Therefore, recombination occurs in the potyvirus genome naturally and frequently and provides new genotypes exposed to host and ecological constraints.

### Phylogenetic Relationships

We initially inferred phylogenetic relationships using the nearly full genomic sequences of the 64 isolates, including all of the recombinants identified in this study. However, the resulting trees had low bootstrap support for some lineages. Therefore, we inferred the tree from the sequences of the 13 non-recombinants. The tree partitioned most of the sequences into the three major consistent genetic groups (data not shown). We then inferred the trees using four partial recombination-cold genomic regions as described above (Figure 5). From the clustering of isolate sequences in each phylogenetic tree, there appear to be five groups (Groups 1, 2, 3, 4, and 5) in nt 110–965 tree (from P1 to HC-Pro protein-coding region), four groups (Groups 1, 2, 3, and 5) in nt 1,520–3,098 tree (from HC-Pro to P3 protein-coding region), four groups (Groups 1, 2, 3, and 5) in nt 3,610–4,523 tree (partial CI protein-coding region), and five groups (Groups 1, 2, 3, 4, and 5) in nt 5,762–9,112 tree (from VPg to CP protein-coding region). Therefore, we found that Group 4 was missing in two out of four phylogenetic trees. Finally, we concluded that there are at least five phylogenetic groups of non-recombinant parental sequences in ScaMV population.

### Similarity and Diversity

Nucleotide sequence identities of ScaMV genomes were calculated by both EMBOSS Needle and Sequence Demarcation Tool (SDT) v1.2. The identities between each isolate calculated by both programs were higher than 80% (data not shown). We also calculated the genetic diversities of ScaMV subpopulations well represented in our data. The genetic diversity values in all protein-coding regions were: polyprotein (0.150±0.014), P1 (0.255±0.017), HC-Pro (0.144±0.006), P3 (0.122±0.005), 6K1 (0.121±0.018), CI (0.126±0.005), 6K2 (0.187±0.025), VPg (0.173±0.009), NIa-Pro (0.188±0.009), NIb (0.181±0.008), and CP (0.106±0.003). Therefore, the genetic diversities of ScaMV were similar in most of the protein-coding regions (0.106–0.188), whereas the P1 protein-coding region is the most diverse, as previously reported for many potyviruses (Montarry et al., 2011; Nigam et al., 2019; Gibbs et al., 2020).

### Timescale Analysis

We evaluated the degree of mutational saturation using the index of substitution saturation, Iss. The estimates of Iss were three to seven times lower than the critical value Iss.c for all datasets (.<0.05). Therefore, there was little saturation across the sequences in the datasets of each of the five protein-coding regions. We then attempted to estimate the evolutionary timescale for ScaMV. However, lack of temporal signals was suggested by the date-randomization test (data not shown) in the ScaMV datasets of four genomic regions we used for phylogenetic analysis (Figure 5). For those reasons, an informative prior distribution to calibrate the clock rate from some earlier studies for potyviruses or from TuMV phylogenetic group viruses is needed. Collecting more ScaMV isolates continuously in Japan would also allow us to observe the measurably evolving population.

### Migration of Non-recombinants and Recombinants

**Figure 6:**
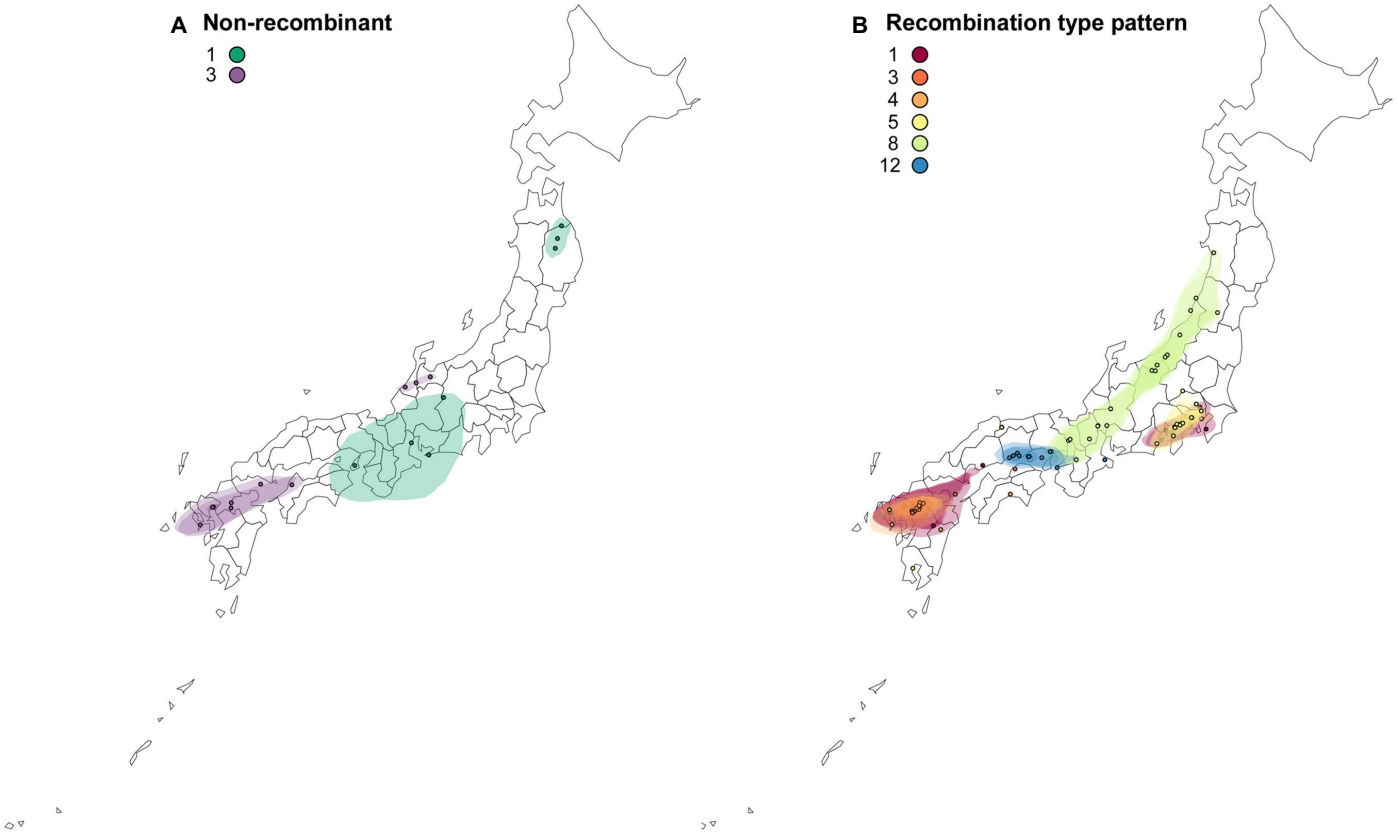
Reconstructed spatial diffusion of ScaMV isolates in Japan for **(A)** non-recombinant and **(B)** recombinant isolates. The point indicated the location of ancestral internal nodes and external tips. The 95% CI regions based on 1,000 trees subsampled from the post-burn-in posterior distribution were shown as colored shadows. The map was obtained from https://gist.github.com/minikomi/4043986

The reconstructed spatial diffusion of ScaMV isolates in Japan was examined for each non-recombinant- and recombination-type pattern (Figure 6). For non-recombinant isolates, distribution of both Group 1 and Group 3 was split into two distinct areas. The distributed area of Group 1 was inferred with broad uncertainty, indicating unsampled isolates of Group 1 scattered more widely around central Honshu. For recombinant isolates, each recombination-type pattern was regionally localized in different parts of Japan. For example, the isolates of recombination-type patterns 1 and 4 were distributed around southern Kanto and northern Kyushu, whereas those of recombination-type patterns 5 and 12 were exclusively distributed, respectively, around southern Kanto and along Seto Inland Sea, which separates Honshu from Shikoku. The isolates of recombination-type pattern 8 were dispersed more broad than the other recombinants but seemed that they were distributed along Japan Sea. This is probably due to the mountain chains that bisect mainland Honshu into the two sides along the Japan Sea and Pacific Ocean.

### Biological Characteristics

ScaMV seemed to be endemic only in Japan and China, although ScaMV has still not been reported in the neighboring countries of South and North Korea. Because this virus infects domestic *Allium* plants in China, we also collected asymptomatic domestic Japanese scallion (*Allium chinense*, also known as Chinese scallion), as well as wild Japanese chives (*Allium schoenoprasum* var. *foliosum*, also known as Asatuki) and wild garlic chives (*Allium tuberosum*, also known as Nira) in Japan, and checked the presence of ScaMV in these plants. However, none of these were infected with ScaMV. Furthermore, we inoculated ScaMV isolates to several *Allium* plants: wild Japanese garlic, leek (*A. ampeloprasum* L. cv. Poireau), onion (*A. cepa* L. cvs. Turbo and Neo-earth), rakkyo (*A. chinensis* G. Don), welsh onion (*A. fistulosum* L. cvs. Koharu, Konatsu, Bannou-konegi, Nebuka-ippon-hutonegi, Ryokushu), Chinese chive (*A. ramosum* L. cv. Ohba-nira), and garlic (*A. sativum* L.). However, we found that Japanese ScaMV only infects wild Japanese garlic plants. Although we were unable to find the virus infection in these plants, continued study is required to monitor the potential for spillovers from wild to domestic *Allium* plants, which would potentially cause an epidemic in Asian countries including Japan.

We found that ScaMV was transmitted by peach aphids, *Myzus persicae* (Sulzer), but not by cotton aphids, *A. gossypii* (data not shown). Thus, aphid transmission tests by several species of aphids will be necessary.

## Conclusion

Our study presents the largest and most detailed epidemiological and evolutionary analyses of a virus from asymptomatic wild plants, highlighting the importance of such studies for exploratory investigation prior to the emergence of pathogens in domestic plants because the wild plants may often serve as reservoirs of viruses. It was a surprise that no mixed infections of different ScaMV isolates were found in wild Japanese garlic, even though many recombination-type patterns were found.

Finally, even though the present study probably captured a representative sample of the genetic diversity of Japanese ScaMV, it is difficult to understand the causes of scattered distribution of the genome types of the two non-recombinants 1 and 3 and one recombinant-type pattern 8 from asymptomatic wild *Allium* plants in Japan (Figure 6). Although we were unable to conclude when and how ScaMV emerged and was introduced from other countries to Japan, it is possible that each genome-type pattern has been introduced several times along different routes because the independent genome types seemed to be non-sequentially distributed in Japan. Otherwise, the ScaMV genome parents recombined in Japan relatively recently and were spread by humans to various districts. To address these hypothesis, further exploratory investigation is needed in the neighboring countries where ScaMV isolates are distributed. Inference of the evolutionary timescale of non-recombinants and recombinants will make these clearer.

## Data Availability Statement

The datasets presented in this study can be found in online repositories. The names of the repository/repositories and accession number(s) can be found in the article/Supplementary Material.

## Author Contributions

KO designed research, wrote the original draft, and was responsible for funding acquisition and the resources. SM, KI, TK, SF, and KO performed research. SK, FG, and KO analyzed data and wrote, reviewed, and edited the manuscript. All authors contributed to the article and approved the submitted version. The authors declare no competing interest.

## Funding

This work was in part funded by Saga University, COC+ program of MEXT, Japan, and the Japanese Society for the Promotion of Science KAKENHI Grant Numbers 24405026, 18KT0092, and 21K05601.

## Conflict of Interest

The authors declare that the research was conducted in the absence of any commercial or financial relationships that could be construed as a potential conflict of interest.

## Publisher’s Note

All claims expressed in this article are solely those of the authors and do not necessarily represent those of their affiliated organizations, or those of the publisher, the editors and the reviewers. Any product that may be evaluated in this article, or claim that may be made by its manufacturer, is not guaranteed or endorsed by the publisher.

## Acknowledgments

We thank Kana Matsuei, Haruka Sato, and Ryosuke Yasaka (Laboratory of Plant Virology, Saga University) for their careful technical assistance. We thank Shinji Kawano (Okinawa Prefecture) and Kazuo Yamashita (Aomori Prefectural Industrial Technology Research Center) for their sample collection. We thank Simon Ho (University of Sydney) for his kindly reading the manuscript before its submission. The genomic sequences of scallion mosaic virus isolates were determined at Laboratory of Plant Virology and Analytical Research Center for Experimental Sciences, Saga University. Computations were partly performed on the National Institute of Genetics (NIG) supercomputer at Research Organization of Information and Systems (ROIS) National Institute of Genetics, Japan. The funders had no role in study design, data collection and analysis, decision to publish, or preparation of the manuscript.

## Footnotes

^1^http://www.ebi.ac.uk/Tools/psa/emboss_needle/

## Supplementary Material

The Supplementary Material for this article can be found online at: https://www.frontiersin.org/articles/10.3389/fmicb. 2021.789596/full#supplementary-material

Supplementary Figure S1 Workflow for full genomic sequencing of scallion mosaic virus. Black lines with arrow heads show the amplified regions by RT-PCR for cloning or direct sequencing for all isolates, and blue lines with arrow heads show the amplified regions to confirm the sequences by direct sequencing for some isolates. Numbers in parenthesis correspond to the genomic positions of Chinese isolate (Chen et al., 2002). NCR; non-coding region, P1; first protein, HC-Pro; helper-component proteinase protein, P3; third protein, 6K1; first 6kDa protein, CI; cylindrical inclusion protein, 6K2; second 6kDa protein, VPg; genome-linked viral protein, NIa-Pro; nuclear inclusion a proteinase protein, NIb; nuclear inclusion b protein, and CP; coat protein. The nucleotide sequences of the genomes of 16 out of 63 isolates were obtained from the cloned plasmids (see Supplementary Table S2).

Supplementary Figure S2 SIMPLOT and GARD analyses of the full genome sequences of scallion mosaic virus. **(A)** Similarity plot with non-recombinants and recombination-type pattern 1 genome sequences were used as the query isolates. Graph of the similarities between the genome sequences of non-recombinants (WOA294, China, and WOEH272) or recombination-type pattern 1 (WOC441) and those of other isolates which belong to other recombination-type patterns. Arrows indicate the position where recombination occurred. The similarities were estimated using SIMPLOT version 3.5.1. Sliding window of 200 nucleotides and step size of 20 nucleotides. **(B)** The best placement of recombination sites (breakpoints) inferred by the algorithm for each number of site considered by GARD.
