# Supplementary material for: Genomic Epidemiology and Evolution of Scallion Mosaic Potyvirus From Asymptomatic Wild Japanese Garlic
Source: Front Microbiol. 2021 Dec 8;12:789596. doi: 10.3389/fmicb.2021.789596 (PMC8692251; doi:10.3389/fmicb.2021.789596)
Supplement: Supplementary Figure S1 — Workflow for full genomic sequencing of scallion mosaic virus. Black lines with arrow heads show the amplified regions by RT-PCR for cloning or direct sequencing for all isolates, and blue lines with arrow heads show the amplified regions to confirm the sequences by direct sequencing for some isolates. Numbers in parenthesis correspond to the genomic positions of Chinese isolate (Chen et al., 2002). NCR; non-coding region, P1; first protein, HC-Pro; helper-component proteinase protein, P3; third protein, 6K1; first 6kDa protein, CI; cylindrical inclusion protein, 6K2; second 6kDa protein, VPg; genome-linked viral protein, NIa-Pro; nuclear inclusion a proteinase protein, NIb; nuclear inclusion b protein, and CP; coat protein. The nucleotide sequences of the genomes of 16 out of 63 isolates were obtained from the cloned plasmids (see Supplementary Table S2). [file Data_Sheet_1.pdf]

SUPPLEMENTARY MATERIAL

Genomic epidemiology and evolution of scallion mosaic potyvirus from asymptomatic wild Japanese garlic

Kazusato Ohshima[1,2,3]*, Shusuke Kawakubo[1], Satoshi Muraoka[1], Fangluan Gao[4], Kanji Ishimaru[1,2,3], Tomoko Kayashima[2,5], and Shinji Fukuda[1,2, 3,6]

[1] Department of Biological Resource Science, Faculty of Agriculture, Saga University, Saga, Japan.

[2] Institute of Wild Onion Science, Saga University, Saga, Japan.

[3] The United Graduate School of Agricultural Sciences, Kagoshima University, Kagoshima, Japan.

[4] Institute of Plant Virology, Fujian Agriculture and Forestry University, Fuzhou, China.

[5] Department of School Education Course, Faculty of Education, Saga University, Saga, Japan.

[6] Saga University Center for Education and Research in Agricultural Innovation, Faculty of Agriculture, Saga University, Saga, Japan.

Correspondence:

Kazusato Ohshima,
ohshimak@cc.saga-u.ac.jp

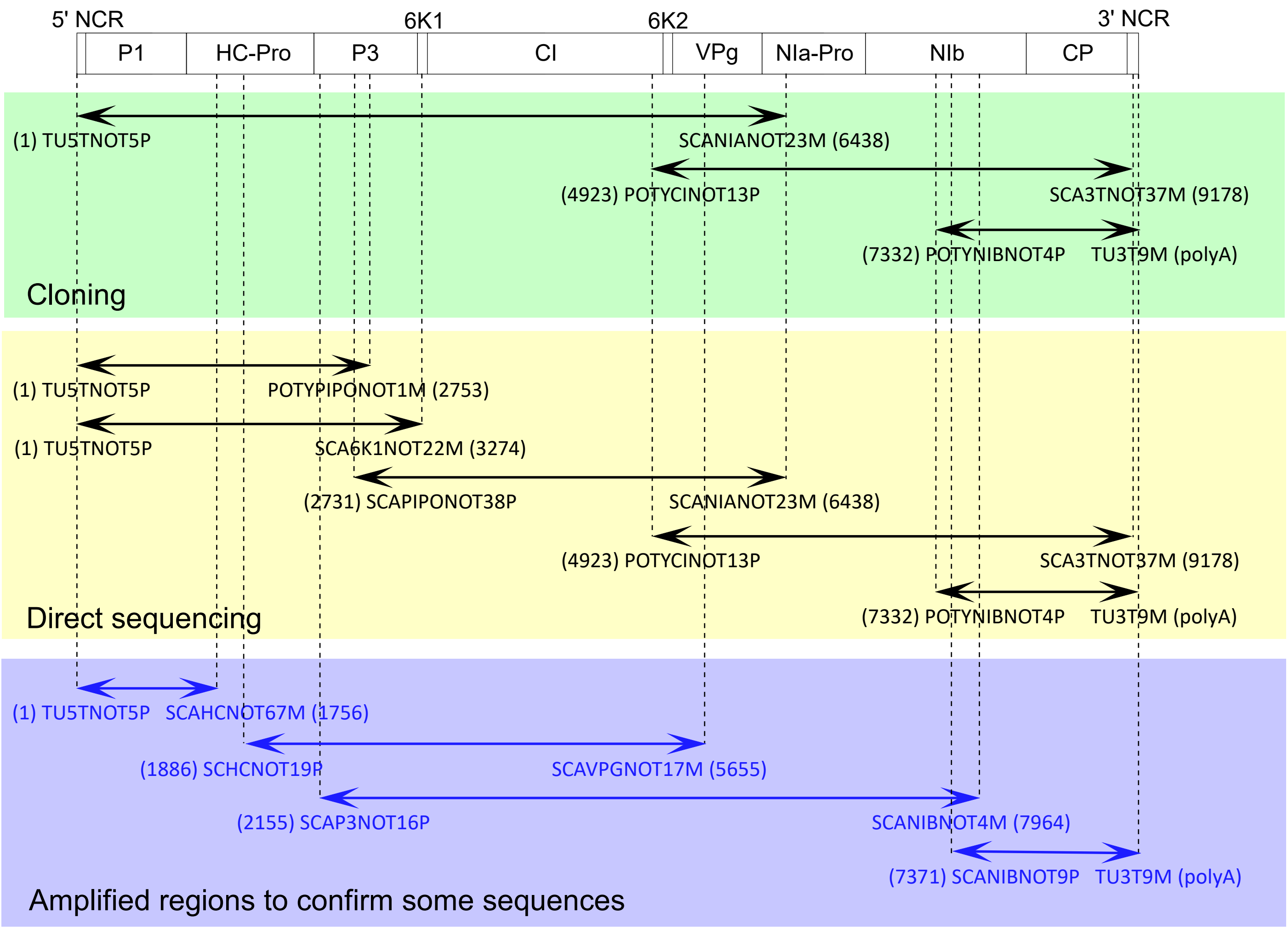

Supplementary Figure 1

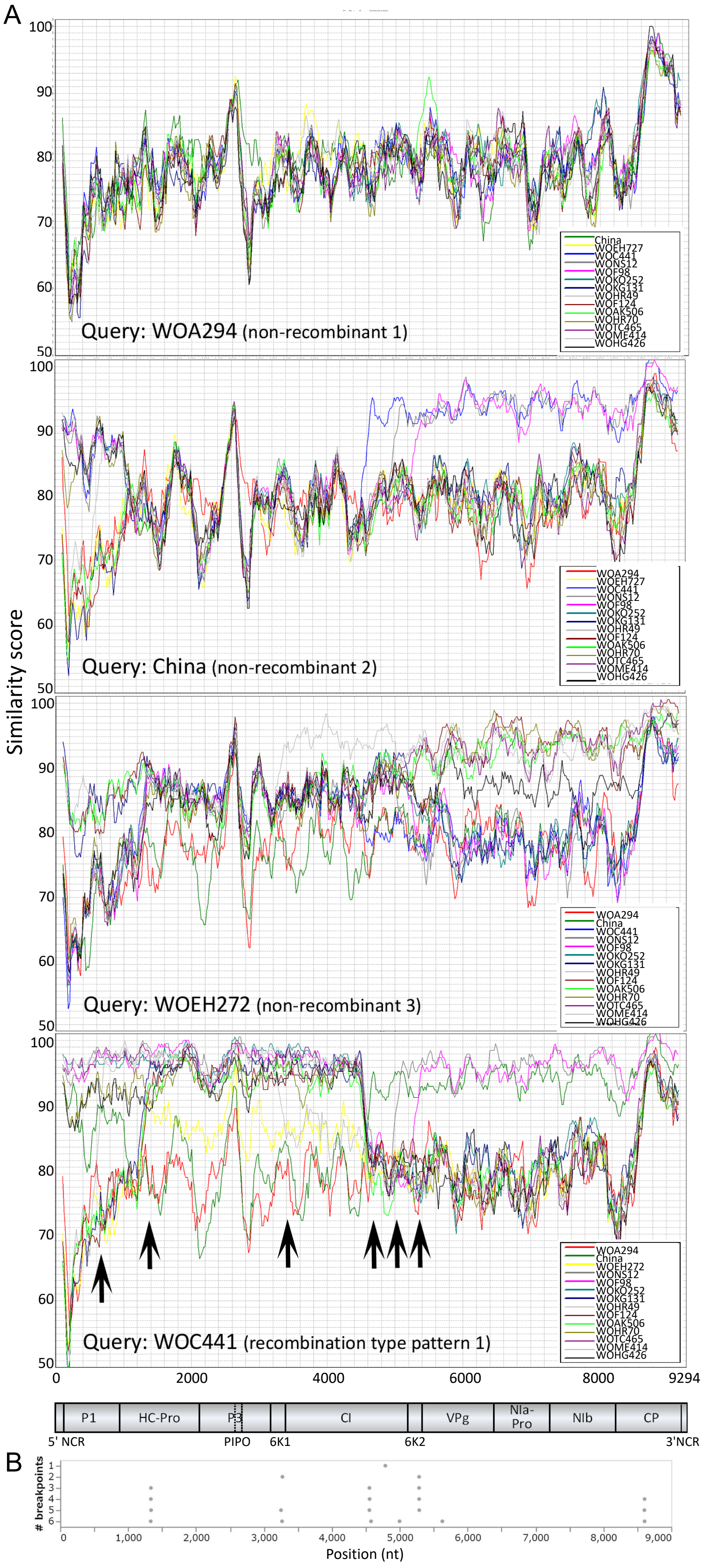

Supplementary Figure 2

Supplementary TABLE 1| Detection of scallion mosaic virus from wild Japanese garlic plants.

District	Plant diagnosed	Plant detected	Incidence of virus infection (%)
Hokkaido	3	0	0
Tohoku	46	6	13
Kanto	42	14	33
Chubu	41	12	29
Kinki	17	9	53
Chugoku	18	9	50
Shikoku	22	3	14
Kyushu	81	20	25
Okinawa	7	0	0
Total	277	73	26

Supplementary TABLE 2| Collection of Japanese garlic plants[1].

Isolate	Location (City, district, prefecture)	Collection date	Potyvirus	Method[2]	Accession no
**Hokkaido district**					
WOHO523	Naganuma-cho274, Yubari-gun, Hokkaido	6 May 2015	ND[3]		
WOHO524	Naganuma-cho274, Yubari-gun, Hokkaido	6 May 2015	ND		
WOHO525	Tachimachi, Hakodateyama, Hakodate, Hokkaido	6 May 2015	ND		
**Tohoku district**					
WOFK319	Higashioeda, Yanagawa-machi, Date, Fukushima	16 June 2014	ND		
WOFK320	Higashioeda, Yanagawa-machi, Date, Fukushima	16 June 2014	ND		
WOFK321	Kunimi-machi, Date-gun, Fukushima	16 June 2014	ND		
WOFK322	Kunimi-machi, Date-gun, Fukushima	16 June 2014	ND		
WOFK486	Meiji307, Itano, Fukushima, Fukushima	23 April 2015	ND		
WOFK488	Akogashima, Atami-machi, Koriyama, Fukushima	23 April 2015	ND		
WOFK491	Yamagata, Yamagata, Inawashiro-machi, Yama-gun, Fukushima	23 April 2015	ND		
WOFK494	Bandai-machi, Aizuwakamatsu, Fukushima	23 April 2015	ND		
WOMY323	Kamiyamanobo, Nobiru, Higashimatsushima, Miyagi	13 June 2014	ND		
WOMY325	Kamiyamanobo, Nobiru, Higashimatsushima, Miyagi	13 June 2014	ND		
WOMY326	Nishiutari, Hamaichi, Higashimatsushima, Miyagi	13 June 2014	ND		
WOMY327	Nishiutari, Hamaichi, Higashimatsushima, Miyagi	13 June 2014	ND		
WOMY328	Yokkaichiba, Shibata-machi, Shibata-gun, Miyagi	15 June 2014	ND		
WOMY329	Yokkaichiba, Shibata-machi, Shibata-gun, Miyagi	15 June 2014	ND		
WOMY478	Shiroishi, Miyagi	22 April 2015	ND		
WOMY480	Funaokatateyama, Shibata-machi, Shibata-gun, Miyagi	22 April 2015	ND		
WOMY482	Sugo, Murata-machi, Shibata-gun, Miyagi	23 April 2015	ND		
WOYA330	Shiromori, Yamagata, Yamagata	12 June 2014	ND		
WOYA333	Shiromori, Yamagata, Yamagata	12 June 2014	ScaMV	DS	This study, LC651516
WOYA335	Kawatoi, Nanyo, Yamagata	12 June 2014	ND		
WOYA504	Ohiwagawa, Tsuruoka, Yamagata	24 April 2015	ScaMV	DS	This study, LC651488
WOIW305	Tamayama-ku, Morioka, Iwate	16 May 2014	ScaMV	DS	This study, LC651463
WOIW308	Kozuya, Ichinohe-machi, Ninohe-gun, Iwate	16 May 2014	ND		
WOIW336	Tachibana, Kitakami, Iwate	15 June 2014	ND		
WOIW340	Yamagishi, Morioka, Iwate	14 June 2014	ND		
WOIW341	Kagoyashiki, Osawa, Takizawa, Iwate	14 June 2014	ND		
WOIW343	Kagoyashiki, Osawa, Takizawa, Iwate	14 June 2014	ND		
WOIW346	Kagoyashiki, Osawa, Takizawa, Iwate	14 June 2014	ND		
WOIW347	Kamiotsukikoji, Ichinoseki, Iwate	13 June 2014	ScaMV	DS (NIb/3'end)	
WOIW348	Kamiotsukikoji, Ichinoseki, Iwate	13 June 2014	ND		
WOIW350	Higashiyama, Morioka, Iwate	14 June 2014	ND		
WOIW515	Uwanonakayanoti, Shizukuishi-machi, Iwate-gun, Iwate	25 April 2015	ND		
WOAK352	Shincho, Yuzawa, Akita	15 June 2014	ND		
WOAK353	Shincho, Yuzawa, Akita	15 June 2014	ND		
WOAK354	Shincho, Yuzawa, Akita	15 June 2014	ND		
WOAK506	Hiroomote, Hiroomote, Akita, Akita	25 April 2015	ScaMV	DS	This study, LC651489
WOA88A	Nanbu-cho, Sannohe-gun, (Kyu-hukuchi), Aomori	8 November 2013	ND		
WOA89A	Nanbu-cho, Sannohe-gun, (Kyu-hukuchi), Aomori	8 November 2013	ND		
WOA294	Nango-ku, Hachinohe, Aomori	16 May 2014	ScaMV	DS	This study, LC651457
WOA295	Nango-ku, Hachinohe, Aomori	16 May 2014	ND		
WOA297	Fukuda, Nanbu-cho, Sannohe-gun, Aomori	16 May 2014	ND		
WOA300	Fukuda, Nanbu-cho, Sannohe-gun, Aomori	16 May 2014	ND		
WOA302	Doshin-cho, Sannohe-machi, Sannohe-gun, Aomori	16 May 2014	ND		
WOA357	Takko-machi, Sannohe-gun, Aomori	8 August 2014	ND		
WOA360	Inakadate-mura, Minamitsugaru-gun, Aomori	8 August 2014	ND		
WOA361	Uwano,, Aomori, Aomori	8 August 2014	ND		
**Kanto district**					
WOKN367	Nurumizu, Atsugi, Kanagawa	4 December 2014	ND		
WOKN372	Terasaka, Oiso-machi, Naka-gun, Kanagawa	5 December 2014	ND		
WOKN375	Yadoriki, Matsuda-machi, Ashigarakami-gun, Kanagawa	6 December 2014	ScaMV	DS	This study, LC651466
WOKN438	Tono-machi, Kawasaki-ku, Kawasaki, Kanagawa	14 March 2015	ScaMV	DS	This study, LC651467
WOKN613	Kawashiri, Midori-ku, Sagamihara, Kanagawa	10 October 2015	ScaMV	DS	This study, LC651468
WOKN614	Kamikasuya, Isehara, Kanagawa	10 October 2015	ND		
WOC111	Shioda-cho, Chuo-ku, Chiba, Chiba	22 November 2013	ND		
WOC113	Shioda-cho, Chuo-ku, Chiba, Chiba	22 November 2013	ND		
WOC115	Ushibukuro, Kisarazu, Chiba	22 November 2013	ND		
WOC117	Ichiibara, Kyonan-machi, Awa-gun, Chiba	22 November 2013	ScaMV	CL (NIb/3'end)	
WOC118	Ichiibara, Kyonan-machi, Awa-gun, Chiba	22 November 2013	ScaMV	DS (NIb/3'end)	
WOC119	Ichiibara, Kyonan-machi, Awa-gun, Chiba	22 November 2013	ND		
WOC441	Kamezawa, Futtu, Chiba	14 March 2015	ScaMV	DS	This study, LC651460
WOC446	Yokoshibahikari-machi, Sanbu-gun, Chiba	14 March 2015	ND		
WOC448	Youkaichiba, Sosa, Chiba	14 March 2015	ND		
WOTK121A	Komagome 2-chome, Toshima-ku, Tokyo	23 November 2013	ScaMV	DS	This study, LC651485
WOTK385	Honmachida, Machida, Tokyo	7 December 2014	ScaMV	DS	This study, LC651486
WOTK389	Utsuki-machi, Hachioji, Tokyo	7 December 2014	ND		
WOTK610	Minamiasakawa-machi, Hachioji, Tokyo	11 October 2015	ND		

[1]Colored row in orange or blue shows that the samples were infected or not infected with scallion mosaic virus, respectively.
[2]DS; Full genomic sequences obtained by direct sequencing of RT-PCR products. CL; Full genomic sequences obtained using cloned plasmids.
NIb/3'end; from the middle of the nuclear inclusion b (NIb) coding region to the 3' end of the viral genomes. NIb / 3' end region indicates where we sequenced.
[3]ND; Scallion mosaic virus was not detected.

Supplementary TABLE 2| Continued.

Isolate	Location (City, district, prefecture)	Collection date	Potyvirus	Method	Accession no
WOST85	Kawaguchi, Saitama	8 July 2013	ND		
WOST86	Kawaguchi, Saitama	8 July 2013	ND		
WOST87A	Kawaguchi, Saitama	19 November 2013	ScaMV	DS	This study, LC651484
WOST87C	Kawaguchi, Saitama	28 November 2013	ScaMV	CL (NIb/3'end)	
WOST108A	Saitama, Saitama	20 November 2013	ND		
WOST110A	Saitama, Saitama	20 November 2013	ScaMV	DS	This study, LC651512
WOST393	Tsuruma, Fujimi, Saitama	7 December 2014	ND		
WOST472	Jinbohara-machi, oji, Kamisato-machi, Saitama	16 March 2015	ScaMV	DS	This study, LC651483
WOST474	Kaminara, Kumagaya, Saitama	16 March 2015	ND		
WOST477	Konosu, Konosu, Saitama	16 March 2015	ND		
WOIB449	Senba-cho, Mito, Ibaraki	15 March 2015	ND		
WOIB450	Kawawada, Mito, Ibaraki	15 March 2015	ND		
WOIB451	Kakurai-cho, Mito, Ibaraki	15 March 2015	ND		
WOIB452	Kamiichibara, Kasama, Ibaraki	15 March 2015	ND		
WOTC453	Onuki, Oji, Motegi-machi, Haga-gun, Tochigi	15 March 2015	ND		
WOTC458	Shimohiraide-machi, Utsunomiya, Tochigi	15 March 2015	ND		
WOTC461	Shizuko, Iwahune-machi, Shimotuga-gun, Tochigi	15 March 2015	ND		
WOTC464	Shimoshibutare-cho, Ashikaga, Tochigi	15 March 2015	ND		
WOTC465	Nishiarai-cho, Ashikaga, Tochigi	15 March 2015	ScaMV	DS	This study, LC651456
WOGU467	Shimotoyooka-machi, Takasaki, Gunma	16 March 2015	ND		
WOGU468	Itahana, Annaka, Gunma	16 March 2015	ND		
WOGU469	Kamitakao, Tomioka, Gunma	16 March 2015	ScaMV	DS (NIb/3'end)	
WOGU471	Shiroishi, Fujioka, Gunma	16 March 2015	ND		
**Chubu district**					
WOAI216	Gonjochi62, Wakami-cho, Tahara, Aichi	18 April 2014	ScaMV	DS	This study, LC651458
WOAI218	Gonjochi62, Wakami-cho, Tahara, Aichi	18 April 2014	ScaMV	DS	This study, LC651459
WOAI220	Gonjochi62, Wakami-cho, Tahara, Aichi	18 April 2014	ND		
WOAI395	Togo-cho, Aichi-gun, Aichi	23 January 2015	ND		
WOAI396	Togo-cho, Aichi-gun, Aichi	23 January 2015	ND		
WOAI397	Oshikamo-cho, Toyota, Aichi	23 January 2015	ND		
WOAI398	Oshikamo-cho, Toyota, Aichi	23 January 2015	ND		
WOAI399	Zoshi, Toyokawa, Aichi	23 January 2015	ND		
WOAI400	Zoshi, Toyokawa, Aichi	23 January 2015	ND		
WOAI401	Nagasawa-cho, Toyokawa, Aichi	23 January 2015	ND		
WOFI554	Kumasaka, Arawa, Fukui	15 May 2015	ND		
WOFI555	Kumano, Obama, Fukui	15 May 2015	ND		
WOFI556	Fukagawa-cho, Tsuruga, Fukui	15 May 2015	ND		
WOFI557	Tenjin, Fukui, Fukui	15 May 2015	ND		
WOIS531	Kawarai-machi, Kanazawa, Ishikawa	15 May 2015	ND		
WOIS532	Shima-machi,Komatsu, Ishikawa	15 May 2015	ScaMV	DS	This study, LC651497
WOIS533A	Uwadana, Shikamachi, Ishikawa	15 May 2015	ND		
WOIS533B	Uwadana, Shikamachi, Ishikawa	15 May 2015	ND		
WONG100C	Usuda, Saku, Nagano	14 November 2013	ND		
WONG226	Nakano, Nagano	28 April 2014	ScaMV	DS	This study, LC651505
WONG290	Iiyama, Nagano	13 May 2014	ScaMV	DS	This study, LC651506
WONG545	Shimadachi, Matsumoto, Nagano	15 May 2015	ND		
WOGF76	Yanagito1-1, Gifu, Gifu	29 March 2013	ScaMV	CL	This study, LC651492
WONI495	Shibata, Niigata	25 April 2015	ND		
WONI498	Miyamae, Sekikawa-mura, Iwafune-gun, Niigata	25 April 2015	ND		
WONI502	Goishi, Murakami, Niigata	25 April 2015	ND		
WONI551	Nishinaka, Itoigawa, Niigata	15 May 2015	ND		
WONI552	Shioyashinden, Zyoetsu, Niigata	15 May 2015	ND		
WONI553	Nagamori, Myoukou, Niigata	15 May 2015	ND		
WOTY539	Goda, Toyama, Toyama	15 May 2015	ScaMV	DS	This study, LC651487
WOTY540	Shiragawa, Himi, Toyama	15 May 2015	ND		
WOGF317	Hida, Gifu	9 June 2014	ND		
WOSH376	Shimotaga, Atami, Shizuoka	5 December 2014	ScaMV	DS (NIb/3'end)	
WOSH377	Ichimichi, Numazu, Shizuoka	6 December 2014	ND		
WOSH378	Ichimichi, Numazu, Shizuoka	6 December 2014	ScaMV	DS	This study, LC651479
WOSH380	Fujimidai, Fuji, Shizuoka	6 December 2014	ND		
WOSH382	Komakado, Gotenba, Shizuoka	6 December 2014	ND		
WOSH404	Echigoshima, Yaizu, Shizuoka	24 January 2015	ND		
WOSH406	Takasaki, Yaizu, Shizuoka	24 January 2015	ScaMV	DS	This study, LC651480
WOSH407	Kitayabe, Shimizu-ku, Shizuoka, Shizuoka	24 January 2015	ScaMV	DS	This study, LC651481
WOSH409	Fuji, Shizuoka	24 January 2015	ScaMV	DS	This study, LC651482
**Kinki district**					
WOHG230	Nakatsu, Hirano-cho, Nishi-ku, Kobe, Hyogo	24 April 2014	ScaMV	DS	This study, LC651493
WOHG234	Zenkai, Ikawadani-cho, Nishi-ku, Kobe, Hyogo	24 April 2014	ND		
WOHG243	HIrotahirota, Minamiawaji, Hyogo	24 April 2014	ScaMV	DS	This study, LC651494
WOHG426	Ohkubo, Akashi, Hyogo	7 March 2015	ScaMV	DS	This study, LC651495
WOKY534	Santanda, Shigetoshi, Sogabe-cho, Kameoka, Kyoto	15 May 2015	ScaMV	DS	This study, LC651518
WOKY535	Furui, Tanba-cho, Funai-gun, Kyoto	15 May 2015	ND		
WOKY536	Kamogawadote, Kyoto, Kyoto	15 May 2015	ND		

Supplementary TABLE 2 | Continued.

Isolate	Location (City, district, prefecture)	Collection date	Potyvirus	Method	Accession no
WONR546	Denen, Gozyo, Nara	15 May 2015	ND		
WONR547	Yanagihara, Gose, Nara	15 May 2015	ScaMV	DS	This study, LC651507
WONR548	Nagahara-cho, Tenri, Nara	15 May 2015	ND		
WOSG538	Yasu, Shiga	15 May 2015	ND		
WOW78	Naka, Wakayama, Wakayama	29 May 2013	ScaMV	CL	This study, LC651515
WOW79	Naka, Wakayama, Wakayama	29 May 2013	ND		
WOW80	Naka, Wakayama, Wakayama	29 May 2013	ND		
WOME414	Kasuga-cho, Matsusaka, Mie	25 January 2015	ScaMV	DS	This study, LC651499
WOME416	Higashioizu-cho, Ise, Mie	25 January 2015	ScaMV	DS	This study, LC651500
WOME419	Hiruda, Tamakicho, Watarai-gun, Mie	25 January 2015	ScaMV	DS	This study, LC651501
**Chugoku district**					
WOT101	Hoki-cho, Saihaku-gun, Tottori	14 November 2013	ScaMV	DS	This study, LC651513
WOT103	Hoki-cho, Saihaku-gun, Tottori	14 November 2013	ScaMV	DS	This study, LC651514
WOOY57	Kume, Kita-ku, Okayama, Okayama	9 March 2013	ND		
WOOY58	Kume, Kita-ku, Okayama, Okayama	9 March 2013	ScaMV	CL	This study, LC651508
WOOY60	Nakanatsukawa, Kita-ku, Okayama, Okayama	9 March 2013	ND		
WOOY64	Nakanatsukawa, Kita-ku, Okayama, Okayama	9 March 2013	ND		
WOOY65	Nakashiima, Kurashiki, Okayama	9 March 2013	ScaMV	CL	This study, LC651509
WOOY67	Konko-cho, Asakuchi, Okayama	9 March 2013	ScaMV	CL	This study, LC651510
WOHR49	Saka-cho, Aki-gun, Hiroshima	8 March 2013	ScaMV	CL	This study, LC651517
WOHR52	Akitsu-cho, Higashihiroshima, Hiroshima	8 March 2013	ScaMV	CL	This study, LC651462
WOHR53	Sunaminishi-machi, Sunaminishi, Mihara, Hiroshima	8 March 2013	ScaMV	CL (NIb/3'end)	
WOHR55	Ozakihon-machi, Onomichi, Hiroshima	8 March 2013	ND		
WOHR56	Ozakihon-machi, Onomichi, Hiroshima	8 March 2013	ND		
WOHR70	Seto-cho, Fukuyama, Hiroshima	9 March 2013	ScaMV	CL	This study, LC651496
WOHR71	Yahatahigashi, Nakaji, Itsukaichi-cho, Saeki-ku, Hiroshima, Hiroshima	10 March 2013	ND		
WOHR72	Yahatahigashi, Nakaji, Itsukaichi-cho, Saeki-ku, Hiroshima, Hiroshima	10 March 2013	ND		
WOHR73	Sagata, Hatsukaichi, Hiroshima	10 March 2013	ND		
WOHR74	Sagata, Hatsukaichi, Hiroshima	10 March 2013	ND		
**Shikoku district**					
WOKO246	Nunoshida, Kochi, Kochi	25 April 2014	ND		
WOKO248	Ikku, Kochi, Kochi	25 April 2014	ND		
WOKO250	Hisaeda, Nankoku, Kochi	25 April 2014	ND		
WOKO252	Osone, Nankoku, Kochi	25 April 2014	ScaMV	DS	This study, LC651498
WOKO254	Osone, Nankoku, Kochi	25 April 2014	ND		
WOKO255	Takaoka-cho, Tosa, Kochi	25 April 2014	ND		
WOKO257	Kitaji, Tosa, Kochi	25 April 2014	ND		
WOKO260	Awa, Susaki, Kochi	25 April 2014	ND		
WOKO262	Kuchigonokawa, Simanto-cho, Takaoka-gun, Kochi	25 April 2014	ND		
WOKO264	Tokonabe, Simanto-cho, Takaoka-gun, Kochi	25 April 2014	ND		
WOKO266	Nishitosaekawa, Shimanto, Kochi	25 April 2014	ND		
WOKO270	Nishitosahage, Shimanto, Kochi	25 April 2014	ND		
WOEH272	Takanoko-machi, Matsuyama, Ehime	26 April 2014	ScaMV	DS	This study, LC651490
WOEH275	Furukawako, Saijo, Ehime	26 April 2014	ND		
WOEH279	Hagyu, Niihama, Ehime	26 April 2014	ND		
WOKW281	Hidono-cho, Zentsuji, Kagawa	26 April 2014	ND		
WOKW282	Yogita-cho, Zentsuji, Kagawa	26 April 2014	ScaMV	DS	This study, LC651469
WOKW284	Kamitakano, Toyonaka-cho, Mitoyo, Kagawa	26 April 2014	ND		
WOKW285	Kamitakano, Toyonaka-cho, Mitoyo, Kagawa	26 April 2014	ND		
WOTS286	Mishma, Anabuki-cho, Mima, Tokushima	26 April 2014	ND		
WOTS600	Nakagirai, Matsushige-cho, Itano-gun, Tokushima	10 March 2015	ND		
WOTS602	Tai, Kaiyou-cho, Kaifu-gun, Tokushima	11 March 2015	ND		
**Kyushu district**					
WON28F	Akinari, Tanushimaru-machi, Kurume, Fukuoka	19 December 2012	ND		
WON29F	Akinari, Tanushimaru-machi, Kurume, Fukuoka	19 December 2012	ND		
WON37F	Miyada, Yoshii-machi, Ukiha, Fukuoka	19 December 2012	ScaMV	CL	This study, LC651477
WOF98	Hara-machi, Yamakawa-machi, Miyama, Fukuoka	14 November 2013	ScaMV	DS	This study, LC651461
WOF124	Hakozaki 6-10-1, Higashi-ku, Fukuoka, Fukuoka	25 December 2013	ScaMV	DS	This study, LC651491
WON26O	Higashioyama, Oyama-machi, Hita, Oita	19 December 2012	ND		
WON30O	Higashioyama, Oyama-machi, Hita, Oita	19 December 2012	ND		
WON31O	Oaza Tomoda, Hita, Oita	19 December 2012	ScaMV	CL	This study, LC651476
WON33O	Kizan-machi, Hita, Oita	19 December 2012	ND		
WON35O	Oaza Ueno, Hitao, Oita	19 December 2012	ND		
WON46O	Kawakami, Yufuin-cho, Yufu, Oita	2 March 2013	ND		
WON47O	Kawakami, Yufuin-cho, Yufu, Oita	2 March 2013	ND		
WON48O	Kawakami, Yufuin-cho, Yufu, Oita	2 March 2013	ND		
WOO195	Sotaro, Saiki, Oita	28 March 2014	ND		
WOO196	Nitahara, Naokawa, Saiki, Oita	28 March 2014	ND		
WOO197	Oaza Ohira, Ume, Saiki, Oita	28 March 2014	ND		
WOO198	Oaza Era, Yayoi, Saiki, Oita	28 March 2014	ND		

Supplementary TABLE 2 | Continued.

Isolate	Location (City, district, prefecture)	Collection date	Potyvirus	Method	Accession no
WONS1	Tenyu 2-chome, Saga, Saga	7 May 2012	ScaMV	CL (NIb/3'end)	
WONS2	Oaza Kanoko, Honjo-machi, Saga, Saga	15 May 2012	ScaMV	CL	This study, LC651503
WONS3	Oaza Kanoko, Honjo-machi, Saga, Saga	15 May 2012	ScaMV	CL (NIb/3'end)	
WONS4	Oaza Suetsugu, Honjo-machi, Saga, Saga	15 May 2012	ScaMV	CL	This study, LC651504
WONS5	Oaza Kanoko, Honjo-machi, Saga, Saga	15 May 2012	ND		
WONS8	Oaza Honjo, Honjo-machi, Saga, Saga	16 May 2012	ND		
WONS9	Tafuse 3-chome, Saga, Saga	16 May 2012	ScaMV	CL	This study, LC651478
WONS11	Hiroenishi, Oaza Kogomori, Honjo-machi, Saga, Saga	18 May 2012	ND		
WONS12	Kyuragi, Kyuragi-machi, Karatsu, Saga	30 November 2012	ScaMV	DS	This study, LC651472
WONS45	Honjo-machi 1,Saga, Saga	22 January 2013	ND		
WONS81	Oaza Shimoizumi, Kuboizumi-machi, Saga, Saga	4 April 2013	ND		
WONS215	Oaza Kanoko, Honjo-machi, Saga, Saga	10 May 2014	ScaMV	DS	This study, LC651511
WON18N	Oura-machi, Nagasaki, Nagasaki	10 December 2012	ND		
WON19N	Tera-machi, Nagasaki, Nagasaki	10 December 2012	ScaMV	CL	This study, LC651502
WON22N	Irabayashi 1-chome, Nagasaki, Nagasaki	10 December 2012	ND		
WON24N	Katafuchi 1-chome, Nagasaki, Nagasaki	10 December 2012	ScaMV	CL	This study, LC651475
WON222	Yoshifuku-cho, Sasebo, Nagasaki	22 April 2014	ND		
WON223	Uwabaru-cho, Sasebo, Nagasaki	22 April 2014	ScaMV	DS	This study, LC651473
WON224	Shimogumigo, Kawatana-cho, Higashisonogi-gun, Nagasaki	22 April 2014	ScaMV	DS	This study, LC651474
WON225	Ishikigo, Kawatana-cho, Higashisonogi-gun, Nagasaki	22 April 2014	ND		
WOK82	Yukino, Kikuchi, Kumamoto	14 April 2013	ND		
WOK83	Shichijo-machi, Kikuchi, Kumamoto	14 April 2013	ND		
WOK84	Shichijo-machi, Kikuchi, Kumamoto	14 April 2013	ScaMV	CL (NIb/3'end)	
WOK92	Oi, Ueki-machi, Kita-ku, Kumamoto, Kumamoto	14 November 2013	ND		
WOK95	Munakata, Yamaga, Kumamoto	14 November 2013	ND		
WOK96	Nagomi-machi, Tamana-gun, Kumamoto	14 November 2013	ND		
WOK136	Terada, Tamana, Kumamoto	5 January 2014	ND		
WOK138	Mono, Ueki-machi, Kita-ku, Kumamoto, Kumamoto	5 January 2014	ND		
WOK140	Sekihokame, Nankan-machi, Tamana-gun, Kumamoto	5 February 2014	ScaMV	DS	This study, LC651464
WOK144	Ohashidori, Yamaga, Kumamoto	5 February 2014	ND		
WOK194	Yanagi, Yamato-cho, Kamimashiki-gun, Kumamoto	27 March 2014	ND		
WOK288	Shmohonjo, Nakamura, Misumi-machi, Uki, Kumamoto	9 May 2014	ND		
WON43M	Marutani-cho, Miyakonojo, Miyazaki	9 January 2013	ND		
WON44M	Marutani-cho, Miyakonojo, Miyazaki	9 January 2013	ND		
WOM145	Nakago, Miyakonojo, Miyazaki	14 February 2014	ND		
WOM146	Nakago, Miyakonojo, Miyazaki	14 February 2014	ND		
WOM147	Nakago, Miyakonojo, Miyazaki	14 February 2014	ND		
WOM148	Naka-machi, Miyakonojo, Miyazaki	14 February 2014	ND		
WOM149	Oza Ohira, Kushima, Miyazaki	14 February 2014	ND		
WOM166	Himegi-cho, Miyakonojo, Miyazaki	14 February 2014	ND		
WOM167	Hiratsuka-cho, Miyakonojo, Miyazaki	14 February 2014	ND		
WOM190	Yamakita, Ebino, Miyazaki	23 February 2014	ND		
WOM191	Yamakita, Ebino, Miyazaki	23 February 2014	ND		
WOM202	Oaza Oshikata, Takachiho, Nishiusuki-gun, Miyazaki	27 March 2014	ND		
WOM206	Oaza Kuwanouchi, Gokase, Nishiusuki-gun, Miyazaki	27 March 2014	ScaMV	DS	This study, LC651470
WOM210	Funeno, Oaza Nanaori, Hinokage, Nishiusuki-gun, Miyazaki	28 March 2014	ScaMV	DS	This study, LC651471
WOM212	Minamikuboyama, Kitakata-machi, Nobeoka, Miyazaki	28 March 2014	ND		
WOM213	Hosomi-machi, Nobeoka, Miyazaki	28 March 2014	ND		
WOM214	Inabazaki-machi, Nobeoka, Miyazaki	28 March 2014	ND		
WOKG130	Shimizu-cho, Kagoshima, Kagoshima	28 December 2013	ND		
WOKG131	Korimoto 1-21-24, Kagoshima, Kagoshima	28 December 2013	ScaMV	CL	This study, LC651465
WOKG150	Koyamada-cho, Kagoshima,Kagoshima	13 February 2014	ND		
WOKG153	Miyama, Higashiichiki-cho, Hioki,Kagoshima	13 February 2014	ND		
WOKG156	Ozato, Ichikikushikino,Kagoshima	13 February 2014	ND		
WOKG159	Shinjo, Tarumizu,Kagoshima	14 February 2014	ND		
WOKG162	Hanaoka-cho, Kanoya,Kagoshima	14 February 2014	ND		
WOKG164	Hishida, Osaki-cho, So-gun,Kagoshima	14 February 2014	ND		
WOKG165	Anraku, Shibushi-cho, Shibushi,Kagoshima	14 February 2014	ND		
WOKG170	Yunoura, Hukiage-cho, Hioki,Kagoshima	15 February 2014	ND		
WOKG174	Hanaze, Kinpo-cho, Minamisatsuma,Kagoshima	15 February 2014	ND		
WOKG177	Nagata, Kawabe-cho, Minamikyushu,Kagoshima	15 February 2014	ND		
WOKG178	Sakurayama-cho, Makusazaki,Kagoshima	15 February 2014	ND		
WOKG182	Kaimonju-cho, Ibusuki,Kagoshima	15 February 2014	ND		
WOKG188	Ikeda, Ibusuki,Kagoshima	15 February 2014	ND		
**Okinawa district**					
WOOK122	Aza Chunjun, Kitanakagusukuson, Nakagami-gun, Okinawa	10 December 2013	ND		
WOOK123	Aza Chunjun, Kitanakagusukuson, Nakagami-gun, Okinawa	10 December 2013	ND		
WOOK221	Ohama, Ishigaki, Okinawa	21 April 2014	ND		
WOOK311	Hateruma, Taketomi-cho, Yaeyama-gun, Okinawa	13 May 2014	ND		
WOOK312	Hateruma, Taketomi-cho, Yaeyama-gun, Okinawa	13 May 2014	ND		
WOOK313	Yonaguni, Yonaguni-cho, Yaeyama-gun, Okinawa	13 May 2014	ND		
WOOK421	Yonaguni, Yonaguni-cho, Yaeyama-gun Okinawa	15 January 2015	ND		

Supplementary TABLE 3| Primers used in this study.

A. Scallion mosaic virus and potyvirus universal primers used for amplifying and sequencing RT-PCR products.

Primer name	Type	Position[1]	Region[2]	Sequence (5' – 3')[3]
TU5TNOT5P	Forward	1-24	5'NCR	GGGGCGGCCGCAAAAAATATAAAAACTCAACACAACA[4]
SCAHCNOT67M	Reverse	1735-1756	HC-Pro	GGGGCGGCCGCGTATGTACATCTTCTGGCCCTC
SCAHCNOT19P	Forward	1886-1905	HC-Pro	GGGGCGGCCGCCTTGACGTGGCAACAGCATG
SCAP3NOT16P	Forward	2155-2175	P3	GGGGCGGCCGCCGACCCACARTTCCATCTCAA
POTYPIPONOT1M	Reverse	2731-2753	P3	GGGGCGGCCGCCGCTGTATCTGCCTCYTAAATC
POTYPIPONOT38P	Forward	2731-2753	P3	GGGGCGGCCGCATATGGGGTGAGAGAGGATTTARRMGGCAGATACRGCG
SCA6K1NOT22M	Reverse	3252-3274	6K1	GGGGCGGCCGCTVRYSACGCAATCRCTMCGTTC
POTYCINOT13P	Forward	4923-4947	CI	GGGGCGGCCGCATATGGGGTGAGAGAGGTWGCMTACACRYTRCARACWGATGT
SCAVPGNOT17M	Reverse	5636-5655	VPg	GGGGCGGCCGCGGATCSACATACCTTACTGC
SCANIANOT23M	Reverse	6416-6438	NIa-Pro	GGGGCGGCCGCTTRGTRSTWATCCAATGTTTCCA
POTYNIBNOT4P	Forward	7332-7353	NIb	GGGGCGGCCGCATATGGGGTGAGAGAGGTNTGYGTNGAYGAYTTYAAYAA
SCANIBNOT9P	Forward	7371-7391	NIb	GGGGCGGCCGCAYTTARTCGCACCTTGGAGTG
SCANIBNOT4M	Reverse	7942-7964	NIb	GGGGCGGCCGCCCTCTTCCAACTTTGGTATCCA
SCA3TNOT37M	Reverse	9159-9178	3'NCR	GGGGCGGCCGCTCTAKRGAGGTGAATACTAACG
TU3T9M	Reverse	Poly A	Poly A	GGGGCGGCCGCTTTTTTTTTTTTTTTTTTTTTTTTTTTTTT

[1]Correspond to the positions in the genome of Chinese isolate (Chen et al., 2002).
[2]NCR; non-coding region, P1; first protein, HC-Pro; helper-component proteinase protein, P3; third protein, 6K1; first 6 kDa protein, CI; cylindrical inclusion protein, 6K2; second 6 kDa protein, VPg; genome-linked viral protein, NIa-Pro; nuclear inclusion a proteinase protein, NIb; nuclear inclusion b protein, and CP; coat protein
[3]R; G+A, Y; C+T, S; G+C, M; A+C, W; A+T, K, T+G, V; A+C+G, D; A+T+G, H; A+T+C, N; A+T+G+C
[4]NotI restriction site; GCGGCCGC (underlined)

B. Scallion mosaic virus primers used for sequencing RT-PCR products.

Primer name[1]	Position[2]	Region [3]	Sequence (5' – 3')[4]	Primer name	Position	Region	Sequence (5' – 3')
SCAP171P	324-345	P1	GTGTGGACCAACTTGTAGGACT	SCACI72P	5097-5117	CI	TYGCRAARGAYAACACAATGG
SCAP144P	326-347	P1	GTGTGGACCAATTAGTAGGGCT	SCACI56P	5099-5120	CI	GCRAARGAYAACACAATGGAGA
SCAP149P	326-347	P1	GCGTAGACCAATTGGTTGGACT	SCA6K230M	5225-5246	6K2	CWAGCGCTCCAAAYTCACGAAC
SCAP159P	326-347	P1	GTGTGGAGAAGCTAATCGGACT	SCAVPG45P	5463-5484	VPg	AGGCTCGTGACAACAAAACAGG
SCAP163P	341-362	P1	GGACTYGTATGTGACATCATGA	SCAVPG18P	5482-5502	VPg	AGGTMGGGARGTGTATGGAGA
SCAP120M	584-604	P1	AAGAAAACCGCTCCAACCTGC	SCAVPG27M	5765-5786	VPg	CTGGTGARCGCCTTATCTGTTG
SCAP128M	584-605	P1	GTAAGAAACCACTCCAACCTGC	SCAVPG43P	5919-5940	VPg	GRCAAACAGGDGAACCACTCCC
SCAP161M	689-710	P1	TCATGCGCACTCGTGCATCAAA	SCAVPG41P	5968-5989	VPg	GCAACTGARTCYGAATTTGCAG
SCAHC40P	758-779	HC-Pro	CGCGGTTTTGGCGTGGTTTCAA	SCANIA82P	6113-6135	NIa-Pro	CRCTRGTGATAACRAATAGGCA
SCAHC24P	855-876	HC-Pro	AAGTGKCAGCACTGCTTTGTCT	SCANIA13M	6266-6286	NIa-Pro	YTGTGTGAAAGGTGGTATGTC
SCAHC62P	930-951	HC-Pro	TAAATCACGAGGGTCAAGCTAG	SCANIA76M	6269-6289	NIa-Pro	GCGYTGTGTRAARGGTGGTAT
SCAHC25M	1007-1028	HC-Pro	TCGCGTGYCTGAATGCTGGATA	SCANIA57M	6416-6438	NIa-Pro	TTDGTGCTTATCCARTGYTTCCA
SCAHC15M	1082-1102	HC-Pro	TCCTTGTAYCTCTGCAAATCC	SCANIA58M	6416-6438	NIa-Pro	TTAGTRCTTATCCAATGYTTCCA
SCAHC21M	1566-1586	HC-Pro	TACTAACGCACGCCTTCGTCA	SCANIA65P	6536-6557	NIa-Pro	GCAAGTTTCCCTGAAGACTTCC
SCAHC12M	1685-1705	HC-Pro	ATCSCCACTATTACCAATCAC	SCANIB47M	6803-6824	NIb	CCCGCACTGTGTGTTTAGTTAC
SCAP334M	2093-2114	P3	CGCCCACTRCGTAATGTTTGAT	SCANIB11M	6854-6874	NIb	TCGTGCTTCATCATGTAACCT
SCAP360M	2097-2112	P3	CACCTACTGCATAGTGTTTGAT	SCANIB81P	7088-7109	NIb	GCYYTAAACATGAAATCRGCAG
SCAP38M	2153-2173	P3	GAGATGGAATTGTGGGTCGCT	SCANIB69P	7090-7111	NIb	GCCTTGAACATGAAATCAGCAG
SCAP314M	2154-2175	P3	TTTGAGATGGAACTGTGGGTC	SCANIB7M	7379-7400	NIb	TCATTCCRACACTCCAAGGTGC
SCAP355P	2273-2295	P3	YTAATAGCWTTYTACAATAGTGG	SCANIB42P	7664-7685	NIb	CCCTCAACAGTYGTTGACAACA
SCAP366P	2278-2299	P3	GTGGCATTCTATAATAGTGGCTC	SCANIB6P	7778-7799	NIb	GGYGATGACTTGTTGATAGGAA
SCAP351P	2488-2509	P3	CCGTATGACACAGCAATGAAGA	SCANIB1P	7893-7914	NIb	AGGCTGACCTCTGGTTCATGTC
SCAP326P	2492-2514	P3	GAYACAGCRATGAAGATGTTGGT	SCANIB35P	7894-7915	NIb	AAAGCCGACCTTTGGTTCATGT
SCAP377M	2644-2665	P3	GGACAAGTTCCCACACGGTGTT	SCANIB5M	7904-7924	NIb	TCCCTTATGTGACATGAACCA
SCAP375P	2732-2753	P3	GATTTAGGAGGCAGATACAGCG	SCANIB73M	7943-7963	NIb	CTCYTCYARCTTTGGTATCCA
SCAP333M	2732-2753	P3	CGCTGTATCTGCCTCCTAAATC	SCANIB46M	7944-7965	NIb	TCCTCCTCYARCTTTGGTATCC
SCAP364P	3075-3096	P3	ATGGAAAGGCGCCAACTTTTGA	SCANIB38P	8021-8042	NIb	GAAGCHATATGCGCAGCRATGA
SCAP329P	3077-3098	P3	GGTAGGGCACCAACKTTTGAAG	SCANIB80P	8137-8157	NIb	AGGMAARGCACCATACATAGC
SCA6K174M	3236-3256	6K1	TCGYTCTRCATCRAACATCAT	SCACP10P	8288-8308	CP	CTGAGGAGTTAGATGCAGGAC
SCACI31P	3432-3453	CI	CRACGTTTGCHATGTGGTGGGC	SCACP2P	8373-8393	CP	TGCAAGAAACTAGTCAGCAAG
SCACI54M	3443-3464	CI	CTAACTGATGAGCCCACCACAT	SCACP36P	8427-8446	CP	ATTCAAGGGACATTCTCTGTGC
SCACI68P	3987-4009	CI	CCAGTGAAAGTGGAGATAGAGG	SCACP70M	8443-8464	CP	CTTTGCTCTTGAGTCGTGGTAC
SCACI53P	4025-4046	CI	AAGAATTCGTACGCGCACAAGG	SCACP39P	8647-8670	CP	GAATGGTTTRATGGTRTGGTGTAT
SCACI48P	4510-4532	CI	ACGGATTGCCCGTAATACCGAA	SCACP3P	8844-8864	CP	TCCCATACATGCCACGGTATG
SCACI32M	4772-4793	CI	GYCTGTCAGGAACRTTTGATCC	SCACP52M	9007-9028	CP	CGTTGCCATCYAAACCAAAAAG
SCACI50P	5033-5054	CI	CAGCAAAYACTGTTTCGTCTTC	SCA3T78M	9301-9324	3'NCR	CTCTAGCRCAYYATCAAAAGTTAG

[1]The last letter of primer, P; forward, M; reverse
[2]Correspond to the positions in the genome of Chinese isolate (Chen et al., 2002).
[3]NCR; non-coding region, P1; first protein, HC-Pro; helper-component proteinase protein, P3; third protein, 6K1; first 6 kDa protein, CI; cylindrical inclusion protein, 6K2; second 6 kDa protein, VPg; genome-linked viral protein, NIa-Pro; nuclear inclusion a proteinase protein, NIb; nuclear inclusion b protein, and CP; coat protein
[4]R; G+A, Y; C+T, S; G+C, M; A+C, W; A+T, K, T+G, V; A+C+G, D; A+T+G, H; A+T+C, N; A+T+G+C

Supplementary TABLE 4| Tentative and clear recombination sites in scallion mosaic virus genomes.

Recombination type pattern[1]	Isolate	Position (nt)[2]	Region[3]	Parental isolate		Recombination detection program[4]	*P*-value[5]
				Major	Minor		
1	WOC441	4556	CI	China	WOM210	RG**B**MCS$_R$	4.24×10^{-93}
	WOHR52	4581	CI	China	WOKN438	**R**GBMCS$_R$	2.12×10^{-110}
	WOM206	4577	CI	China	WOM210	**R**GBMCS$_R$	3.60×10^{-93}
	WONS9	4630	CI	China	WOM210	**R**GBMCS$_R$	5.25×10^{-117}
	WON224	4556	CI	China	WOM210	RG**B**MCS$_R$	3.82×10^{-90}
	WOSH407	4556	CI	China	WOM210	RG**B**MCS$_R$	4.71×10^{-97}
	WOSH409	4581	CI	China	WOM210	**R**GBMCS$_R$	5.22×10^{-89}
	WOST87A	4556	CI	China	WOM210	RG**B**MCS$_R$	3.02×10^{-93}
2	WONS12	5008	CI	China	WOM210	**R**GBMCS$_R$	1.16×10^{-82}
3	WOF98	5290	6K2	China	WOM210	**R**GBMCS$_R$	1.73×10^{-96}
	WOK140	5290	6K2	China	WON210	**R**GBMCS$_R$	6.17×10^{-102}
	WOKW282	5214	6K2	China	WON210	RG**B**MCS$_R$	4.15×10^{-96}
4	WOKO252	5879	VPg	China	WOOY58	RGBMC**S$_R$**	6.85×10^{-51}
	WOKN438	5879	VPg	China	WOOY58	RGBMC**S$_R$**	1.26×10^{-51}
	WOKN613	5879	VPg	China	WOOY58	RGBMC**S$_R$**	2.80×10^{-52}
	WOM210	5879	VPg	China	WOOY58	RGBMC**S$_R$**	7.64×10^{-50}
	WON24N	5945	VPg	China	WOOY58	RGBMC**S$_R$**	4.30×10^{-51}
	WON37F	5879	VPg	China	WOOY58	RGBMC**S$_R$**	5.19×10^{-49}
	WON31O	5875	VPg	China	WOOY58	RGBMC**S$_R$**	1.79×10^{-49}
	WON223	5879	VPg	China	WOOY58	RGBMC**S$_R$**	9.54×10^{-50}
5	WOKG131	5948	VPg	China	WOOY58	RGBMC**S$_R$**	2.99×10^{-48}
	WOKN375	5879	VPg	China	WOOY58	RGBMC**S$_R$**	1.61×10^{-49}
	WOSH378	5879	VPg	China	WOOY58	RGBMC**S$_R$**	3.93×10^{-48}
	WOSH406	5879	VPg	China	WOOY58	RGBMC**S$_R$**	6.79×10^{-50}
	WOST472	5879	VPg	China	WOOY58	RGBMC**S$_R$**	1.25×10^{-50}
	WOTK121A	5879	VPg	China	WOOY58	RGBMC**S$_R$**	7.22×10^{-50}
	WOTK385	5879	VPg	China	WOOY58	RGBMC**S$_R$**	3.48×10^{-51}
6	WOHR49	609, 4997	P1, CI	WONS4	WOKO252	RG**B**MCS$_R$	1.40×10^{-111}
7	WOF124	5453	6K2	Unknown (China)	WON24N	**R**GBMCS$_R$	1.54×10^{-53}
8	WOAK506	4556	CI	Unknown (China)	WON24N	RG**B**MCS$_R$	1.48×10^{-54}
	WOGF76	5151	CI	Unknown (China)	WON24N	RGBMC**S$_R$**	1.35×10^{-44}
	WOHG230	4499	CI	Unknown (China)	WON24N	RG**B**MCS$_R$	6.76×10^{-58}
	WOKY534	5146	CI	Unknown (China)	WON24N	**R**GBMCS$_R$	6.57×10^{-49}
	WONG226	4556	CI	Unknown (China)	WON24N	RG**B**MCS$_R$	1.09×10^{-59}
	WONG290	4979	CI	Unknown (China)	WON24N	**R**GBMCS$_R$	8.23×10^{-46}
	WONR547	5424	CI	Unknown (China)	WON24N	RG**B**MCS$_R$	7.40×10^{-57}
	WOST110A	5151	CI	Unknown (China)	WON24N	RGBMC**S$_R$**	1.84×10^{-45}
	WOT101	4556	CI	Unknown (China)	WON24N	RG**B**MCS$_R$	2.40×10^{-55}
	WOT103	5151	CI	Unknown (China)	WON24N	RGBMC**S$_R$**	1.64×10^{-46}
	WOYA333	5142	CI	Unknown (China)	WON24N	RG**B**MCS$_R$	1.77×10^{-51}
	WOYA504	5146	CI	Unknown (China)	WON24N	**R**GBMCS$_R$	5.55×10^{-48}
9	WOHR70	1088	HC-Pro	WOIS532	China	**R**BMCS$_R$	1.88×10^{-73}
		5670	VPg	WOKO252	WOEH272	**R**GBMCS$_R$	3.69×10^{-72}
10	WOTC465	4871	CI	WON19N	WOKO252	RG**B**MCS$_R$	3.80×10^{-102}
11	WOME414	3294	6K1/CI	WON19N	WOKO252	**R**GBMCS$_R$	5.45×10^{-126}
	WOME416	3311	CI	WON19N	WOKO252	**R**GBMCS$_R$	1.16×10^{-124}
12	WOHG426	UD	UD	WOIS532	WOM210	**R**GBMCS$_R$	4.33×10^{-28}
	WOME419	UD	UD	WOIS532	WOM210	**R**GBMCS$_R$	3.81×10^{-32}
	WOOY58	1349	HC-Pro	Unknown (China)	WOIS532	**R**GBMCS$_R$	5.64×10^{-25}
	WOOY65	1336	HC-Pro	WOIS532	China	**B**CS$_R$	2.89×10^{-6}
	WOOY67	1349	HC-Pro	WOIS532	China	RBMC**S$_R$**	7.40×10^{-5}
	WOW78	UD	UD	WOIS532	WOM210	**R**BMS$_R$	8.99×10^{-5}

[1]Recombination type patterns are listed in Figure 4.
[2]Recombination sites detected in the ScaMV genomes by the recombination detection programs. The nucleotide positions show locations in the aligned genomic sequences of 9294 nucleotide long in the present study. UD; Undetermined.
[3]P1; fist protein, HC-Pro; helper-component proteinase protein, 6K1; first 6 kDa protein, CI; cylindrical inclusion protein, 6K2; second 6 kDa protein, and VPg; genome-linked viral protein
[4]Recombinant isolates identified by the recombination detection programs: R (RDP), G (GENECONV), B (BOOTSCAN), M (MAXCHI), C (CHIMAERA) and S_R (SISCAN) programs in RDP4. The analyses were done using default settings and a Bonferroni-corrected *P*-value cut-off of 0.01 in RDP4 version 100. The programs of those *P*-value calculated in equal and smaller than 1.0×10^{-4} are listed and the programs those showed the smallest *P*-value are shown in bold type and underlined.
[5]The smallest *P*-value are shown.

SUPPLEMENTARY REFERENCES

Chen, J., Zheng, H. Y., Chen, J. P., and Adams, M. J. (2002). Characterisation of a potyvirus and a potexvirus from Chinese scallion. Arch. Virol. 147, 683-693. doi: 10.1007/s007050200018
